# Organelle stress in NLRP3 inflammasome: a central mediator of neurodegenerative diseases

**DOI:** 10.1186/s13024-025-00922-0

**Published:** 2026-01-03

**Authors:** Huachao Jia, Lianghua Ma, Jinyue Liu, Menglin Gao, Xuxin Liang, Fan Zhang, Yanzi Gao, Mingyan Liu, Wanwei Jiang, Minjie Wei, Xin Zhong

**Affiliations:** 1 School of Pharmacy, China Medical University, Shenyang, Liaoning 110122 China; 2 Department of Radiation Oncology, The First Hospital of China Medical University, Shenyang, Liaoning 110801 China; 3 Department of Anesthesiology, Affiliated Zhongshan Hospital of Dalian University, Dalian, Liaoning 116001 China; 4 Liaoning Medical Diagnosis and Treatment Center, Shenyang, Liaoning 110167 China

**Keywords:** NLRP3 inflammasome, Organelle stress, Neurodegenerative diseases, Organelle interactions, Therapeutic targets, Mitochondria, Endoplasmic reticulum stress, Lysosomal dysfunction

## Abstract

Organelle stress and NLRP3 inflammasome activation have been established as pivotal contributors to inflammatory responses and play a significant role in the pathogenesis of neurodegenerative diseases (NDDs), including Alzheimer’s disease (AD) and Parkinson’s disease (PD). However, the involvement of multiple organelles and their interorganellar signaling in NLRP3 inflammasome activation has not yet been systematically elucidated. The causal mechanisms by which these organelles promote NLRP3 activation and subsequently drive the progression of NDDs remain unclear. Furthermore, therapeutic interventions targeting this pathway require urgent and comprehensive investigation. This article comprehensively reviews the mechanisms by which organelle-derived stress signals, such as mitochondrial reactive oxygen species (mtROS), cathepsin B (CTSB), and calcium ions (Ca^2+^), induce NLRP3 inflammasome activation. It further examines the pivotal role of organelle-specific NLRP3 localization and post-translational modifications in the activation of the NLRP3 inflammasome. Finally, we outline NLRP3-mediated inflammatory processes in NDDs, focusing on events directly induced by organelle stress and interactions. These processes are not merely consequences of NDD progression but also critical factors accelerating disease deterioration. Potential therapeutic strategies targeting the organelle-NLRP3 axis are proposed, which may provide novel theoretical foundations and promising avenues for the prevention and treatment of NDDs.

**Graphical Abstract:**

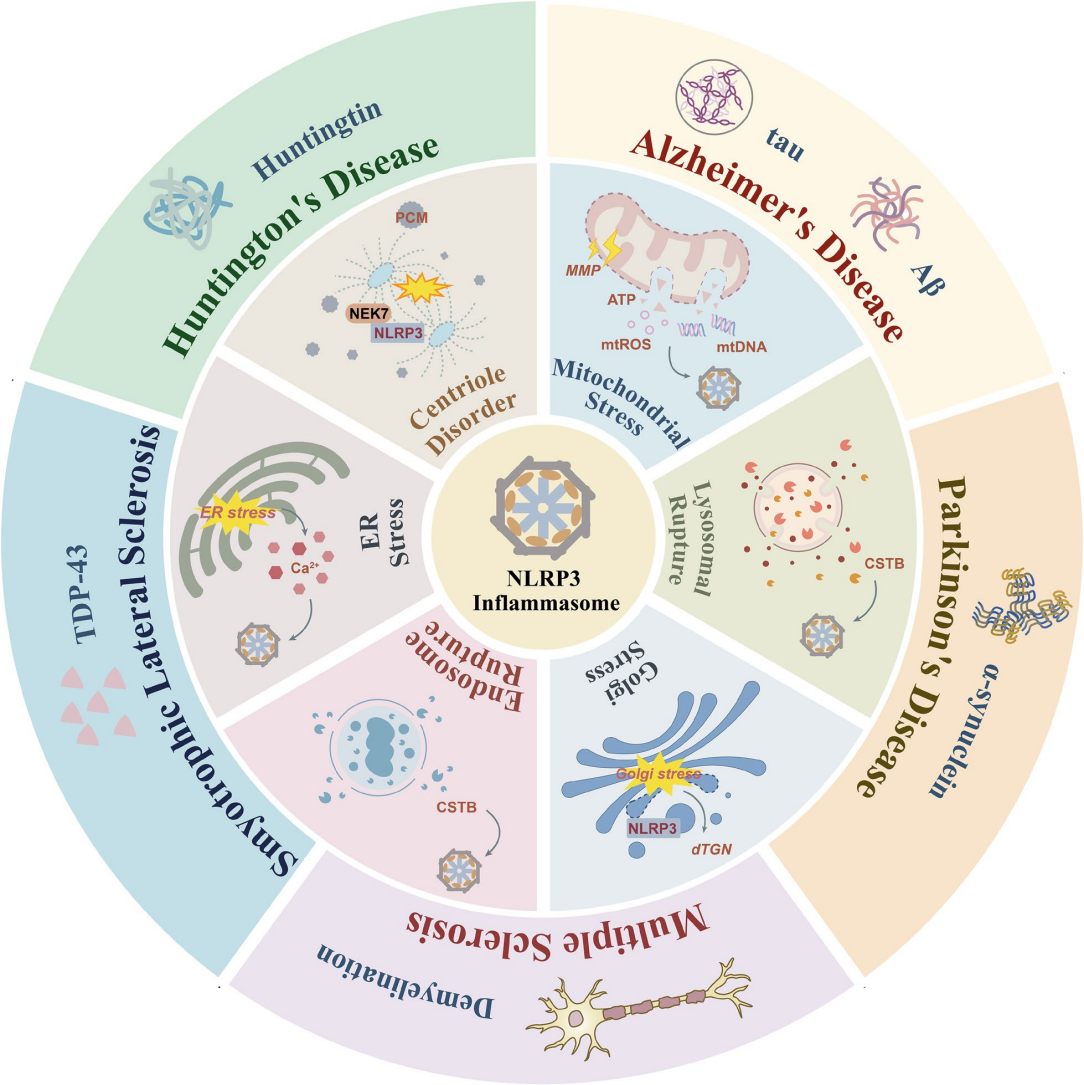

## Introduction

The NOD-like receptor family pyrin domain-containing 3 (NLRP3) inflammasome is a key component of the innate immune system. It consists of three core components: NLRP3, apoptosis-associated speck-like protein containing CARD (ASC), and pro-cysteine aspartate-specific protease-1 (pro-caspase-1) [1]. As an intracellular pattern recognition receptor, NLRP3 functions as a sensor for both pathogen-associated molecular patterns (PAMPs) and damage-associated molecular patterns (DAMPs). Upon sensing these activation signals, NLRP3 transitions from its self-inhibited state and undergoes oligomerization through the assistance of the adaptor protein NIMA-related kinase 7 (NEK7) to form the inflammasome complex. This core structure facilitates the helical oligomerization of the adaptor protein ASC, thereby creating a signaling platform for the activation of cysteine aspartate-specific protease-1 (caspase-1). Activated caspase-1 subsequently cleaves gasdermin D (GSDMD) to induce pyroptosis, and simultaneously processes the inactive precursors of interleukin-1β (lL-1β) and interleukin-18 (lL-18) into their mature forms, which are then secreted to initiate an inflammatory response [2, 3]. Recent studies have suggested that, in addition to classical ion fluxes such as K^+^ efflux and Ca^2+^ influx, mitochondrial reactive oxygen species (mtROS), mitochondrial DNA (mtDNA), and cathepsin B (CTSB) are signaling molecules that actively participate in and amplify the activation of the NLRP3 inflammasome [4, 5]. These molecules originate from distinct organelles and are frequently associated with cellular events including mitochondrial metabolic dysfunction, dysregulation of the unfolded protein response (UPR), and alterations in lysosomal membrane permeability [6–8]. Therefore, during inflammatory responses, organelles may not merely function as signal carriers; rather, their stress responses likely serve as critical bridges linking upstream inflammatory stimuli to NLRP3 inflammasome activation. These organelle-mediated processes provide essential prerequisites for NLRP3 inflammasome activation through structural remodeling, the release of signaling molecules, and related mechanisms. Furthermore, dynamic inter-organelle interactions, such as mitochondria-endoplasmic reticulum contact sites (MERCs) and endoplasmic reticulum-Golgi trafficking, can form intricate regulatory networks, contributing to the spatiotemporal regulation of NLRP3 activation [9, 10].

Importantly, NLRP3 is widely expressed in neurons and glial cells and functions as a key intracellular sensor for various stress or injury signals, enabling the formation of NLRP3 inflammasomes. In neurodegenerative diseases (NDDs), which are progressive disorders primarily characterized by neuronal dysfunction, synaptic loss, and abnormal protein aggregation, neuroinflammation has garnered increasing attention. Aberrant activation of the NLRP3 inflammasome is considered a central hub linking cellular damage to inflammatory responses [11]. Notably, the NLRP3 inflammasome intersects with multiple core pathological pathways in NDDs. Specifically, in Alzheimer’s disease (AD), amyloid-β (Aβ) deposition can directly trigger NLRP3 activation in microglia of APP/PS1 mice, whereas genetic ablation of NLRP3 enhances glutamine and glutamate metabolism and improves mitochondrial function [12]. In Parkinson’s disease (PD), mitochondrial dysfunction amplifies the NLRP3 inflammasome-dependent pro-inflammatory cascade in microglia, thereby exacerbating dopaminergic neuron degeneration [13]. Thus, metabolic and oxidative signals arising from organelle stress, such as mitochondrial impairment and lysosomal damage, are likely key upstream events driving NLRP3 inflammasome activation [14–18]. In turn, the resulting inflammatory response promotes protein misfolding, impairs autophagic clearance, and amplifies cellular stress signals, establishing a self-sustaining vicious cycle leading to progressive neuronal loss [19, 20]. Inhibition of the NLRP3-caspase-1 axis has been shown to effectively reduce Aβ deposition in AD models and mitigate neuronal degeneration in PD and amyotrophic lateral sclerosis (ALS) models [21–23]. These findings suggest that the interplay between the NLRP3 inflammasome and organelle stress may offer novel theoretical insights and potential therapeutic strategies for the prevention and treatment of neuroinflammation in NDDs.

Although the association between organelle stress and the NLRP3 inflammasome in NDDs has been elucidated, several core issues regarding its mechanism of action remain unresolved. Specifically, how does the dynamic subcellular localization of NLRP3 regulate its activation? How do different organelles coordinate to modulate the activation of NLRP3? What roles do these processes play in the pathogenesis and progression of NDDs? In light of these questions, this review focuses on the stress responses of key organelles such as mitochondria and the ER. It systematically analyzes the molecular mechanisms by which these organelles activate the NLRP3 inflammasome through multiple pathways, including the release of signal molecules like mtDNA, regulation of NLRP3 subcellular localization, and post-translational modifications (PTMs) of proteins. Furthermore, it highlights the critical role of organelle interactions in inflammatory signal transduction. Additionally, characteristic pathological events in NDDs, such as abnormal tau protein aggregation, can establish a positive feedback loop of neuroinflammation and accelerate disease progression by activating the organelle stress-NLRP3 signaling axis. Based on these insights, we summarize novel therapeutic strategies targeting organelle homeostasis and the NLRP3 signaling axis, aiming to provide a theoretical foundation for the investigation of neuroinflammatory mechanisms and drug development for NDDs.

## The activation mechanism of the NLRP3 inflammasome

As a core component of the NLRP3 inflammasome, NLRP3 maintains an autoinhibited conformation in the resting state through intramolecular interactions between its NACHT domain and the leucine-rich repeat (LRR) domain. This structural arrangement prevents exposure of the PYD domain, thereby preserving the protein in an inactive state [24]. Full assembly and activation of functional NLRP3 inflammasomes require two sequential signaling events in the canonical pathway: priming and activation.

During the priming phase, pattern recognition receptors (PRRs), including Toll-like receptors (TLRs), are activated upon recognition of PAMPs or DAMPs. For instance, extracellular lipopolysaccharide (LPS) triggers the nuclear factor-κB (NF-κB) signaling pathway via the TLR4-myeloid differentiation primary response gene 88 (MYD88)/TIR domain-containing adapter-inducing interferon-β (TRIF) pathways, thereby promoting the transcriptional upregulation of the NF-κB signaling cascade. This promotes the transcriptional upregulation of NLRP3, pro-interleukin-1β (pro-IL-1β), and pro-interleukin-18 (pro-IL-18), which provides the necessary molecular components for subsequent inflammasome assembly [25, 26]. Concurrently, the NLRP3 protein undergoes various PTMs, including phosphorylation and ubiquitination, which stabilize it in a self-inhibited yet signaling-competent conformation [27]. These PTMs exert continuous regulatory effects on both the initiation and resolution of inflammatory responses [27].

The activation of the NLRP3 inflammasome is initiated by a diverse array of PAMPs, including LPS, peptidoglycan, and nigericin, as well as DAMPs such as ATP and cholesterol crystals (CCs), α-synuclein (α-syn), Aβ, alum, and silica [3]. These stimuli trigger NLRP3 inflammasome activation through interconnected downstream events, including dysregulated ion flux across membranes and organelle dysfunction [28]. Notably, agents such as LPS, nigericin, ATP, and monosodium urate (MSU) induce mitochondrial damage, leading to the release of mtROS and mtDNA, which serve as critical initiators of the NLRP3-mediated inflammatory response [29, 30]. Furthermore, mtROS facilitate NLRP3–NEK7 interaction and ASC oligomerization by promoting the translocation of chloride intracellular channels (CLICs) to the plasma membrane, thereby inducing Cl^-^ efflux [31, 32]. Concurrently, endoplasmic reticulum stress (ERS) exacerbates inflammatory signaling via calcium signal crosstalk, with elevated intracellular Ca^2 +^ levels directly enhancing the assembly of NLRP3 inflammasome components [33, 34]. Additionally, bacterial pore-forming toxins and extracellular ATP (eATP) bind to the P2X7 receptor (P2X7R), a plasma membrane receptor, resulting in K^+^ efflux that drives NLRP3 activation [35–37]. While K^+^ efflux, along with alum, cholesterol crystals, and silica, contributes to lysosomal membrane disruption and CTSB release, synergistically promoting NLRP3 inflammasome activation [38–42]. During this process, Na^+^ influx serves solely as an auxiliary regulatory factor [33]. Ultimately, NEK7 serves as a critical sensor of K^+^ efflux, binds to the LRR domain of NLRP3, relieves its autoinhibitory conformation, and facilitates structural rearrangements in the NACHT domain that enable oligomerization [43]. Subsequently, ASC is recruited through PYD-PYD homotypic interactions, which further bridges pro-caspase-1 via CARD-CARD interactions to assemble and form the complete NLRP3 inflammasome [43]. Activated caspase-1 not only cleaves pro-IL-1β and pro-IL-18 into mature inflammatory cytokines but also cleaves GSDMD to generate its N-terminal fragment, which forms membrane pores. This process induces pyroptosis, thereby establishing a detrimental inflammatory feedback loop [44–46].

The non-canonical inflammasome activation pathway is primarily mediated by human caspase-4 and caspase-5, along with their murine ortholog caspase-11, with the core mechanism involving the direct recognition of LPS in the cytoplasm. Extracellular LPS activates TLR4 and induces caspase-11 expression via TRIF-dependent type I interferon (IFN-I) responses, particularly through the interferon-β (IFNβ)-interferon-α/β receptor 1 (IFNAR) signaling axis [26, 47]. Notably, caspase-11 activation, specifically its proteolytic processing, does not depend on pattern recognition receptors such as TLRs, nor is it entirely independent of NLRP3 and ASC. Rather, caspase-4/5/11 themselves function as intracellular LPS sensors [48, 49]. Upon infection, intracellular Gram-negative bacteria such as Brucella are disrupted by guanylate-binding proteins (GBPs), leading to the release of LPS into the cytosol [50, 51]. Caspase-4/5/11 then directly bind to the lipid A of LPS via their CARD domains, which induces self-oligomerization and subsequent protease activation [52, 53]. Activated caspase-11 specifically cleaves GSDMD, generating GSDMD-N-terminal domain (GSDMD-NT) that triggers pyroptosis and facilitates the secretion of IL-1β [54, 55]. Additionally, caspase-11 promotes K^+^ efflux and forms membrane pores through GSDMD-NT, enabling the release of mtDNA [56, 57]. These downstream events further activate the NLRP3 inflammasome, indicating that the non-canonical pathway can converge with the canonical inflammasome pathway, thereby jointly amplifying the inflammatory response. Moreover, human caspase-4 is capable of cleaving both pro-IL-18 and GSDMD, allowing for IL-18 release independently of the canonical inflammasome and caspase-1 [58, 59]. Inhibition of caspase-4/5 has been shown to suppress IL-1β production [60], suggesting the existence of a compensatory interplay between the non-canonical and canonical inflammasome pathways in humans, which enhances the complexity and adaptability of the immune response.

**Fig. 1:**
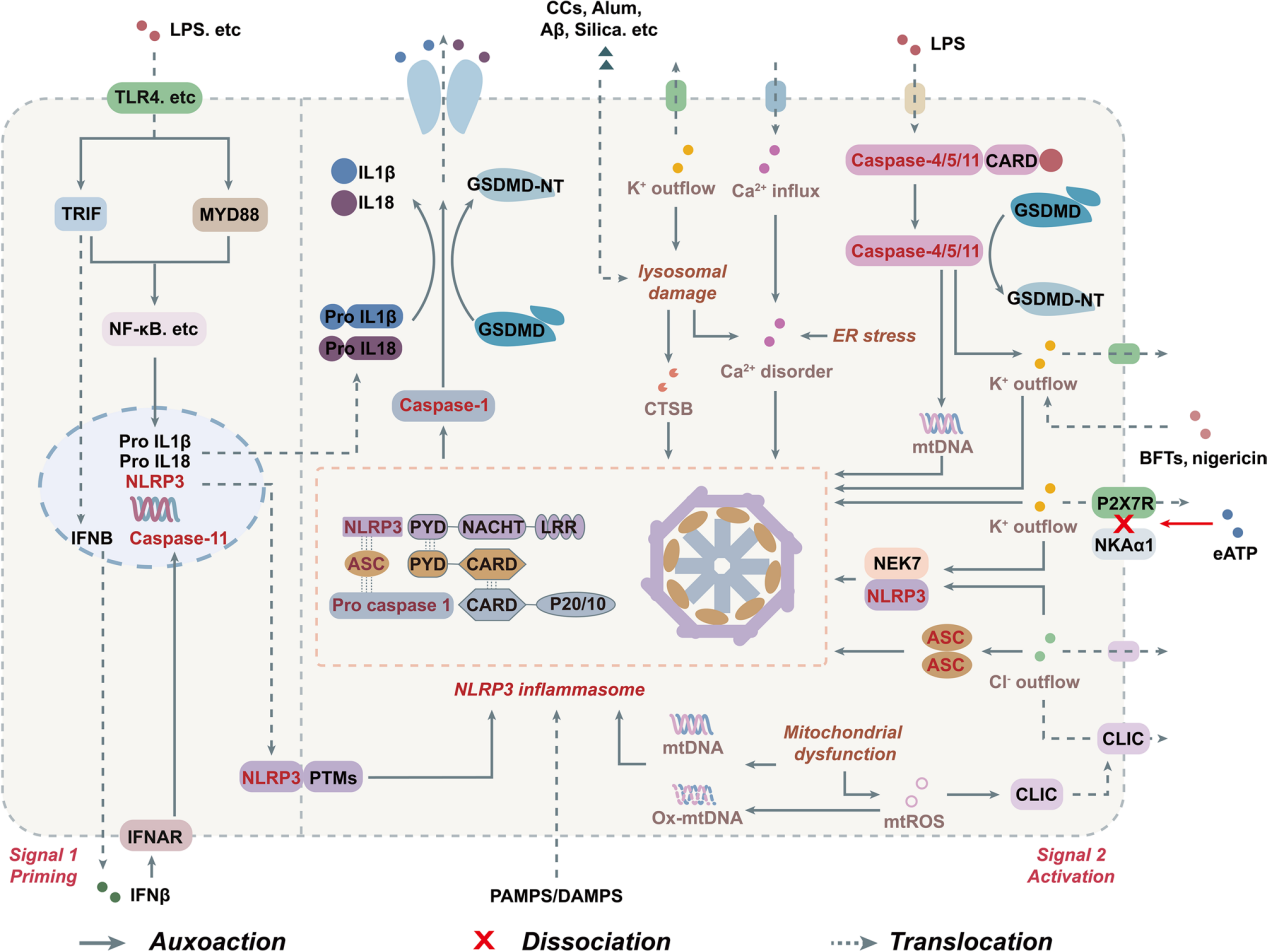
The priming and activation of the NLRP3 inflammasome. In the priming signal (signal 1), pattern recognition receptors such as TLR4 recognize PAMPs or DAMPs, including LPS, leading to the activation of downstream signaling pathways such as NF-κB. This activation promotes the transcriptional upregulation of NLRP3, pro-IL-1β, pro-IL-18, and caspase-11. In the activation signal (signal 2), diverse stimuli including ATP and microbial toxins, trigger a range of cellular disturbances such as ion flux imbalances and mitochondrial dysfunction, which facilitate the assembly of NLRP3 with ASC and pro-caspase-1 into a functional inflammasome complex. The resulting activation of caspase-1 enables the proteolytic cleavage of pro-IL-1β, pro-IL-18, and GSDMD, thereby promoting the maturation and secretion of inflammatory cytokines and inducing pyroptosis. In the non-canonical pathway, caspase-4/5/11 can directly bind intracellular LPS. This interaction not only induces pyroptosis through direct cleavage of GSDMD but also indirectly activates the NLRP3 inflammasome via mechanisms such as potassium ion efflux, thus synergistically amplifying the inflammatory response initiated by the canonical pathway. Abbreviations: caspase-1, cysteine aspartate-specific protease-1; CTSB, cathepsin B; ER, endoplasmic reticulum; CLICs, chloride intracellular channels; GSDMD, gasdermin D; GSDMD-NT, GSDMD N-terminal domain; IFNAR, interferon-α/β receptor 1; IFNβ, IFN interferon-β; mtDNA, mitochondrial DNA; mtROS, mitochondrial reactive oxygen species; MYD88, TLR4-myeloid differentiation primary response gene 88; NEK7, NIMA-related kinase 7; NF-κB, nuclear factor-κB; NKAα1, Na⁺/K⁺-ATPase α1; NLRP3, the NOD-like receptor family pyrin domain-containing 3; PTMs, post-translational modifications; P2X7R, P2X purinergic receptor 7; TLR4, Toll like receptor 4; TRIF, TIR domain-containing adapter-inducing interferon-β

In summary, NLRP3 inflammasome activation is a tightly regulated, multistep process. The canonical pathway involves priming and activation, which are triggered by stimuli such as ionic flux and mitochondrial damage, leading to NEK7-mediated oligomerization and caspase-1-dependent pyroptosis. In contrast, the non-canonical pathway is initiated by intracellular LPS, which directly activates caspase-4/5/11. These caspases cleave GSDMD, inducing K^+^ efflux, which subsequently converges with the canonical pathway (Fig. 1). Notably, both pathways are closely associated with dysfunction of multiple organelles, highlighting that organelle homeostasis serves as a central regulatory node in NLRP3 inflammasome activation.

## The regulatory network of organelle stress and the NLRP3 inflammasome

**Fig. 2:**
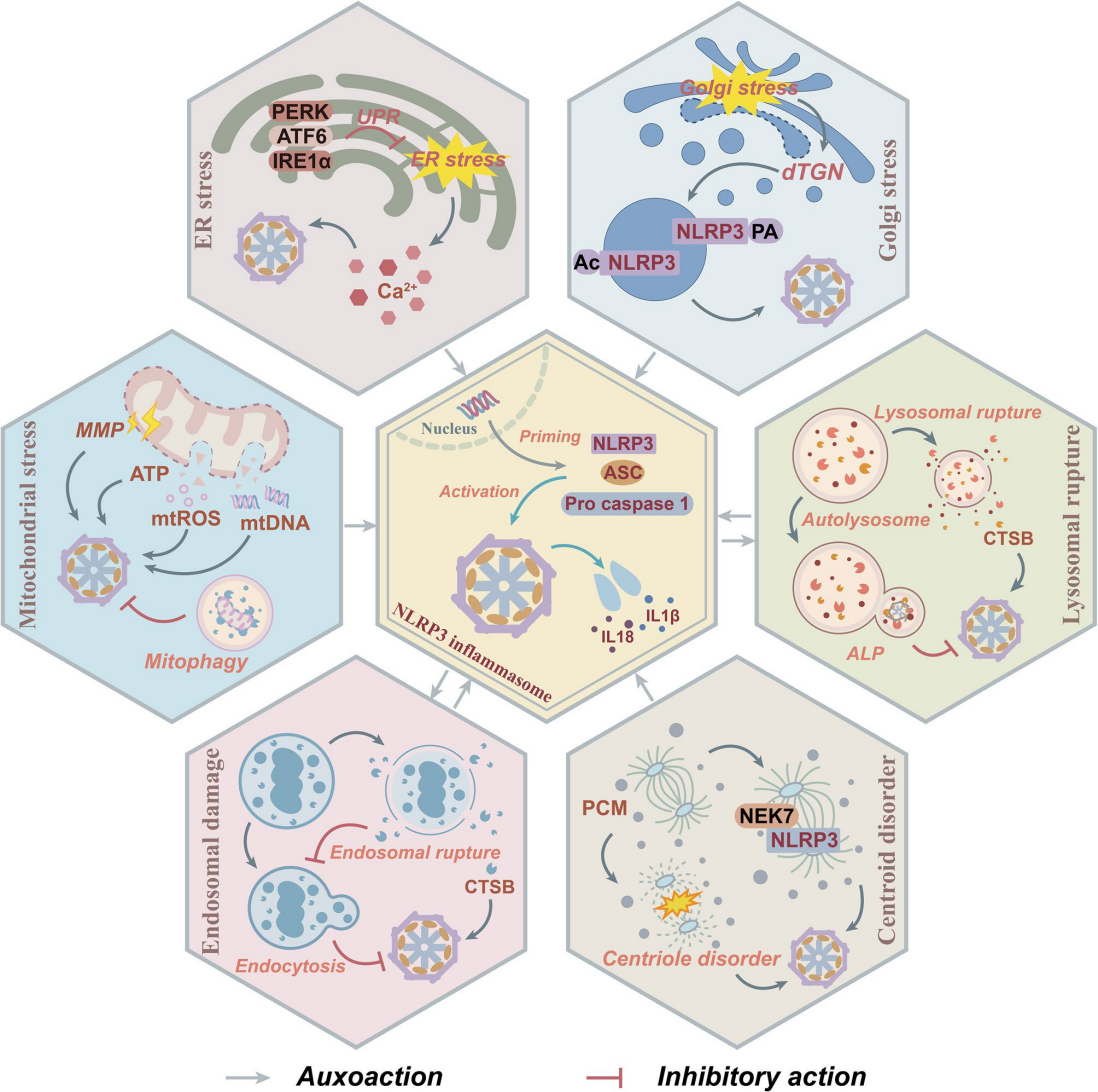
The regulatory mechanism between organelle stress and NLRP3 inflammasome. NLRP3, ASC, and pro-caspase-1 are assembled into the NLRP3 inflammasome via interactions between the PYD and CARD domains, thereby facilitating the maturation of IL-1β/IL-18 and inflammatory responses. This process is closely associated with the activation of cellular stress signaling pathways. Upon mitochondrial stress, a decrease in MMP leads to the release of signal molecules such as ATP, mtROS, and mtDNA, which subsequently activate the NLRP3 inflammasome. The UPR mediated by PERK, ATF6, and IRE1α inhibits ERS-induced Ca^2+^ release and activates the NLRP3 inflammasome. Golgi apparatus stress promotes the formation of dTGN, recruits NLRP3 to dTGN, and undergoes acetylation and palmitoylation, thereby enhancing the assembly of the NLRP3 inflammasome. Lysosomal rupture releases CTSB to activate the NLRP3 inflammasome; however, ALP can suppress its generation. Endosome rupture also releases CTSB to activate the NLRP3 inflammasome while inhibiting endosome-mediated endocytosis, whereas endosome-mediated endocytosis antagonizes the formation of the NLRP3 inflammasome. Both centrosome dysfunction and NEK7 promote the localization of NLRP3 along microtubules and activate the NLRP3 inflammasome. Abbreviations: ALP, autophagy-lysosome pathway; ASC, apoptosis-associated speck-like protein containing card; ATF6, activating transcription factor 6; CTSB, cathepsin B; dTGN, dispersed trans-Golgi network; ER, endoplasmic reticulum; IL-1β, interleukin-1β; IL-18, interleukin-18; IRE1α, inositol-requiring eenzyme 1α; MMP, mitochondrial membrane potential; mtDNA, mitochondrial DNA; mtROS, mitochondrial reactive oxygen species; NEK7, NIMA-related kinase 7; NLRP3, the NOD-like receptor family pyrin domain-containing 3; PCM, pericentriolar material; PERK, protein kinase R-like ER kinase; UPR, unfolded protein response

Recent studies have demonstrated that organelles, including mitochondria, ER, Golgi apparatus, endosome, lysosome, and centrosome, can collectively regulate the assembly and activation of the NLRP3 inflammasome via structural damage, release of DAMPs, and mediation of NLRP3 localization. For instance, mtROS and mtDNA, released as a result of mitochondrial dysfunction, directly activate NLRP3 via oxidative stress and the cyclic GMP-AMP synthase (cGAS)-stimulator of interferon genes (STING) pathway [61]. ERS induces NLRP3 activation through the UPR and calcium signaling dysregulation. The Golgi apparatus finely tunes the inflammatory activity of NLRP3 by modulating its subcellular localization and PTMs. Furthermore, the release of CTSB, triggered by changes in lysosomal membrane permeability and endosomal injury, along with the structural remodeling of NLRP3 mediated by the centrosome-associated protein NEK7, highlights the multi-dimensional regulatory role of organelle stress in neuroinflammation (Fig. 2). Consequently, an in-depth investigation into the roles of stress events in various organelles in driving NLRP3 inflammasome activation through spatial regulations and molecular interactions is crucial for elucidating the precise regulatory mechanisms underlying NLRP3 inflammasome function.

### The regulation of NLRP3 inflammasome by mitochondria

Mitochondria, serving as the central hub for cellular energy metabolism and oxidative stress regulation, exert critical regulatory functions in the NLRP3 inflammasome activation process [62]. First, mitochondrial stress induces the release of signaling molecules such as mtROS and mtDNA, which can directly or indirectly activate the NLRP3 inflammasome [63–65]. Second, mitochondrial dynamics, mediated by fusion and fission processes, maintain organelle structural integrity and provide a scaffold for the assembly of inflammatory signaling complexes [66]. Third, metabolic reprogramming within mitochondria can bidirectionally regulate inflammation through alterations in cellular energy balance and redox homeostasis [67, 68]. This functional shift from an “energy factory” to an “inflammatory signal integrator” unveils a novel mechanism by which mitochondrial stress regulates innate immunity.

#### The role of mitochondria as a source of stress signals

Mitochondrial stress drives the activation of NLRP3 inflammasomes through multiple pathways by releasing key signaling molecules such as mtROS and mtDNA.

As the central signaling hub, mtROS not only activate the cGAS-STING pathway, leading to the phosphorylation of TANK-binding kinase 1 (TBK1) and interferon regulatory factor 3 (IRF3), but also induce a IFN-I response [69]. Concurrently, mtROS establish a positive feedback loop by inhibiting the silent information regulator 3 (SIRT3)-superoxide dismutase 2 (SOD2) antioxidant system, thereby promoting their own accumulation. This exacerbates mitochondrial dysfunction, leading to a reduction in mitochondrial membrane potential (MMP) and the release of oxidized-mitochondrial DNA (Ox-mtDNA), ultimately triggering NLRP3 activation [70]. The activation of the nuclear factor erythroid 2-related factor 2 (NRF2)/heme oxygenase-1 (HO-1) pathway has been shown to counteract this process [71]. Moreover, mtROS can activate NLRP3 via multiple non-classical mechanisms. On one hand, mtROS may induce NLRP3 independently of K^+^ efflux by inhibiting endogenous pathways involving enzymes such as quinone oxidoreductase NQO2 and mitochondrial complex I [72]. On the other hand, in the caspase-4/11-mediated non-canonical inflammasome pathway, GSDMD-NT pore formation further promotes ROS upregulation mediated by mitochondrial complex II, thereby triggering NLRP3 activation. This not only amplifies inflammatory signaling but also establishes a positive feedback loop that exacerbates pore formation and mitochondrial damage [73].

Notably, mtDNA, particularly Ox-mtDNA, not only amplifies inflammation through the cGAS-STING pathway, but also activates NLRP3 through both the canonical caspase-1 pathway and the non-canonical caspase-11 pathway [57, 61, 74]. This immunostimulatory effect may be attributed to the evolutionary homology between mtDNA and bacterial DNA, which renders mtDNA more readily recognizable by innate immune sensors [75]. Of particular significance, Ox-mtDNA appears to possess a stronger potential for inflammatory activation, as evidenced by the PYD of NLRP3 preferentially binding to Ox-mtDNA. Moreover, cytoplasmic Ox-mtDNA can directly bind to NLRP3, exerting a more pronounced effect on the NLRP3 inflammatory response [76, 77]. However, even NLRP3 lacking the PYD domain can still bind to mtDNA and mediate inflammatory responses [77]. This indicates that mtDNA recognition mechanism exhibits a level of complexity that is not entirely dependent on structural factors. Collectively, these findings underscore the pivotal role of mitochondrial stress signals in regulating neuroinflammation and reveal the intricate nature of their underlying molecular networks.

#### The role of mitochondria in structural scaffolding and metabolic integration

Mitochondrial dynamics play a critical role in the fine-tuning of the NLRP3 inflammatory response through the regulation of mitochondrial fusion and fission equilibrium. Studies have demonstrated that upregulating mitochondrial fusion proteins, specifically mitofusin 1 (MFN1) and optic atrophy protein 1 (OPA1), or inhibiting the activity of dynamin-related protein 1 (DRP1), which mediates mitochondrial fission, can substantially attenuate the activation of the NLRP3 inflammasome [78]. This underscores the importance of mitochondrial structural integrity as a regulatory mechanism in inflammation. Furthermore, mitochondria-localized metabolic proteins indirectly modulate NLRP3 activity by influencing cellular energy homeostasis and redox status. For instance, family with sequence similarity 3 member A (FAM3A) functions as a pivotal regulator of mitochondrial metabolism. It activates the phosphatidylinositol 3-kinase (PI3K)/protein kinase B (AKT)/NRF2 signaling axis, thereby preventing mtROS accumulation. Conversely, downregulation of FAM3A results in excessive mtROS production, leading to NLRP3-dependent pyroptosis [79]. Similarly, phospholipase A2 group VII (PLA2G7), a central node in lipid metabolism, promotes lysophosphatidylcholine (LPC) production, which induces proton leakage and elevates mtROS levels [80]. Notably, deficiency of PLA2G7 reduces fatty acid oxidation and suppresses downstream caspase-1 activation, highlighting the significance of lipid metabolic reprogramming in inflammasome assembly. Collectively, these findings demonstrate that mitochondria integrate multiple signaling networks through their structural plasticity and metabolic regulatory functions, enabling precise control over NLRP3-mediated inflammatory responses.

#### The metabolic regulatory role of mitochondria

Mitochondrial metabolic reprogramming achieves bidirectional regulation of the NLRP3 inflammatory response through the dynamic balance of energy metabolism and redox states. With regard to pro-inflammatory mechanisms, aerobic glycolysis can promote the activation of NLRP3 inflammasomes through multiple pathways. First, upregulation of the metabolic enzymes hexokinase II (HK2) and pyruvate kinase M2 (PKM2) has been shown to directly facilitate NLRP3 inflammasome assembly and IL-1β secretion [81, 82]. Second, LPS enhances glucose uptake by upregulating glucose transporter 1 (GLUT1), thereby providing a substrate for glycolysis. While inhibition of this pathway has been demonstrated to effectively attenuate the NLRP3-mediated inflammatory response [83]. Notably, deletion of polynucleotide phosphorylase 1 (PNPT1) specifically increases glycolytic activity and mitochondrial antiviral signaling protein (MAVS) expression, leading to NLRP3 inflammasome activation independently of NF-κB signaling [84]. In contrast, OXPHOS and enhancement of the tricarboxylic acid (TCA) cycle exert anti-inflammatory effects. Specifically, γ-aminobutyric acid (GABA) enhances flavin adenine dinucleotide (FAD)-lysine specific demethylase 1 (LSD1) signaling pathway, which regulates histone demethylation of downstream genes and reduces the succinylation levels of mitochondrial proteins. This enhancement promotes OXPHOS and consequently inhibits the formation of NLRP3 inflammasomes [85]. Conversely, inhibition of mitochondrial complexes I-III disrupts OXPHOS integrity and promotes NLRP3 inflammasome activation [86]. Collectively, these findings indicate that the status of mitochondrial energy metabolism serves as a central regulatory signal for inflammation, and the preservation of its functional integrity is essential for maintaining immune homeostasis.

### The regulation of NLRP3 inflammasome by ER

As a central organelle integral to protein synthesis, lipid metabolism, and calcium storage, the maintenance of ER homeostasis is fundamental to cellular function [87]. The accumulation of excessive unfolded or misfolded proteins within the ER can induce ERS, leading to protein overload and calcium homeostasis imbalance [87, 88]. ERS not only constitutes a core component of the cellular stress response but also modulates NLRP3 inflammasome activity through multiple molecular mechanisms.

#### Bidirectional regulatory role of UPR and GRP78

During the early phase of ERS, cells initiate the UPR to restore physiological equilibrium. The UPR is mediated by three ER-resident sensors, including inositol-requiring enzyme 1α (IRE1α), protein kinase R-like endoplasmic reticulum kinase (PERK), and activating transcription factor 6 (ATF6) [89]. This process is regulated by the ER molecular chaperone glucose-regulated protein 78 (GRP78/BiP). Under basal conditions, GRP78 binds to these sensors, maintaining them in an inactive state. Upon ERS, GRP78 preferentially interacts with misfolded proteins, releasing IRE1α, PERK, and ATF6, thereby activating downstream UPR signaling to reestablish proteostasis [90–93]. However, when UPR exceeds a specified limit, persistent accumulation of misfolded proteins leads to elevated GRP78 expression, whose function shifts from protective regulation to pro-inflammatory signal transduction. By activating the NF-κB-NLRP3 pathway to trigger inflammatory responses, GRP78 thereby emerges as a pivotal signaling hub linking protein folding defects to NLRP3-driven inflammation [94].

#### Inflammatory regulatory network mediated by the UPR signaling

Importantly, the three branches of the UPR differentially regulate ERS-associated inflammation, forming a sophisticated regulatory network. The IRE1α-XBP1s signaling axis upregulates thioredoxin-interacting protein (TXNIP) expression, which, under ROS-mediated oxidation, dissociates from thioredoxin (Trx) and directly interacts with NLRP3, facilitating inflammasome assembly. This mechanism underscores ROS as a key intermediary connecting ERS to NLRP3 activation [95, 96]. Concurrently, the PERK pathway phosphorylates eIF2α, leading to increased expression of activating transcription factor 4 (ATF4) and subsequent induction of C/EBP homologous protein (CHOP), a key mediator of cellular stress [97]. Notably, CHOP not only promotes the expression of apoptosis-related genes but also enhances caspase-4/11 activity, thereby directly coupling UPR signaling to the non-canonical inflammasome pathway [98, 99]. Additionally, upon dissociation from GRP78, ATF6 translocates to the Golgi apparatus via COPII vesicles in response to ERS signals [100, 101]. Following proteolytic activation, ATF6 exacerbates the NLRP3-mediated inflammatory response through the ATF6-CHOP and ATF6–P2X7R axes [102, 103]. Thus, ERS contributes to the synergistic activation of the NLRP3 inflammasome through multiple pathways, including GRP78 transformation, IRE1α-ROS-TXNIP coupling, PERK/ATF4-CHOP-mediated apoptotic signaling, downstream pathways of ATF6, and activation of non-canonical inflammasome pathways. These pathways collectively form an integrated regulatory network that drives excessive inflammatory responses.

#### Direct and indirect mechanisms of ERS-mediated NLRP3 activation

Moreover, as a pivotal hub in inflammatory regulation, ERS modulates NLRP3 inflammasome activation through both direct and indirect pathways. In the direct pathway, ERS induces the dissociation of TXNIP or activates the TLR4–MYD88-NF-κB signaling cascade, thereby triggering the NLRP3 inflammatory response [104–106]. Moreover, various physical and chemical stimuli, such as bacterial endotoxin (BE), arsenic exposure, and microgravity, can induce sustained ERS, leading to direct activation of the NLRP3 inflammasome [107–109]. Regarding indirect mechanisms, ERS-induced calcium release from the ER leads to intracellular Ca^2 +^ mobilization, which may secondarily activate the NLRP3 inflammasome via mitochondrial dysfunction and other stress-related processes [110–112]. Concurrently, ERS-induced ROS accumulation and impaired NRF2-mediated antioxidant defense collectively establish an oxidative stress microenvironment favorable for caspase-11 and NLRP3 activation [113]. Notably, ERS can promote NLRP3 activation by initiating non-canonical inflammasome pathways. For instance, following cleavage of GRP78 by the subtilase cytotoxin (SubAB) toxin, the PERK/IRE1α signaling pathway may upregulate the expression of caspase-11 [114]. While inhibition of signaling pathways such as nuclear factor of activated T Cells, cytoplasmic, calcineurin-dependent 1 (NFATc1) and TLR4-NF-κB has been shown to alleviate ERS, thereby indirectly suppressing NLRP3 inflammasome activation [115, 116]. These findings collectively indicate that ERS orchestrates a complex regulatory network involving multiple signaling cascades that converge on NLRP3 inflammasome activation.

### The regulation of NLRP3 inflammasome by Golgi apparatus

The Golgi apparatus, serving as the key hub for intracellular protein processing and signal transduction, consists of cis-Golgi, medial-Golgi, and trans-Golgi cisternae [117]. The Golgi apparatus, particularly the trans-Golgi network (TGN), provides an essential structural platform for the activation of the NLRP3 inflammasome. The TGN not only acts as a critical localization site for NLRP3 but also directly modulates its inflammatory activity via PTMs [118, 119].

#### Golgi apparatus serves as the structural foundation for NLRP3 recruitment

NLRP3 specifically localizes to the TGN, and its activators such as Nigericin and Gramicidin can induce structural remodeling of TGN, leading to its disassembly into dispersed trans-Golgi network (dTGN) vesicles [119, 120]. The formation of dTGN not only serves as a marker of Golgi stress but also represents a critical structure that determines the assembly of the NLRP3 inflammasome. Specifically, the oligomerization of the PYD domain of the ASC protein relies on the co-localization of NLRP3 and dTGN. Moreover, dTGN functions as a scaffold for the interaction between NLRP3 and ASC, thereby facilitating the downstream inflammatory signaling cascade [119]. This process may be mediated by the phosphatidylinositol 4-phosphate (PI4P)- GOLPH3-myosin XVIIIA (MYO18A) complex [121]. Moreover, the targeted trafficking of NLRP3 to dTGN is mediated by the coordinated action of multiple key regulatory proteins, including PI4P, sterol regulatory element-binding protein cleavage-activating protein (SCAP), and glycogen synthase kinase 3 β (GSK3β), which facilitate targeted transport through direct binding to NLRP3 [119, 122, 123]. Additionally, small hydrophobic protein (SH) may assist in NLRP3 entry into the Golgi apparatus by mediating channel formation in the Golgi membrane [124].

The Golgi apparatus and ERS do not exhibit a simple upstream-downstream linear relationship during NLRP3 activation, rather, they form an interdependent and mutually reinforcing signaling network. On one hand, ERS-induced UPR leads to the translocation of ATF6 to the Golgi apparatus for proteolytic activation, thereby initiating transcription of downstream target genes [92]. Furthermore, disruption of ER-Golgi trafficking has been shown to inhibit NLRP3 inflammasome activation, indicating that ERS signaling may be dependent on the function of the Golgi apparatus [125, 126]. On the other hand, NLRP3 can still be activated under conditions where the Golgi apparatus undergoes fragmentation in the absence of detectable ERS [127]. This evidence suggests that the dTGN, which serves as a critical assembly platform for NLRP3, can be formed independently of ERS. Therefore, there may exist a positive feedback loop between ERS and the Golgi apparatus. Specifically, ERS promotes Golgi fragmentation, while impaired Golgi function exacerbates ERS due to disruptions in protein processing and trafficking, resulting in mutual amplification of inflammatory signals. Furthermore, the functional dynamics of regulatory proteins such as immunity-related GTPase M (IRGM) also reflect this complexity [128]. ASC resides at the Golgi with IRGM at homeostasis, where they interact with NLRP3 and ASC domains to suppress inflammasome oligomerization and assembly [129, 130]. Importantly, the physical interaction between IRGM and ASC is dependent on the presence of NLRP3 [130]. Upon NLRP3 activation, ASC dissociates from IRGM, leading to its release from the Golgi, while residual IRGM promotes Golgi fragmentation through phosphorylation of Golgi specific brefeldin A resistance factor 1 (GBF1), thereby amplifying the inflammatory response [129]. This transition suggests a dual regulatory role for IRGM during inflammation: in the basal state, it stabilizes the Golgi structure and exerts anti-inflammatory effects; however, upon inflammatory stimulation, IRGM shifts toward promoting Golgi disassembly and amplifying the inflammatory response.

#### Golgi apparatus mediates PTMs in NLRP3

Studies have demonstrated that specific PTMs, such as palmitoylation and acetylation, can enhance the activation of the NLRP3 inflammasome, whereas ubiquitination and deSUMOylation suppress its activation [131, 132]. Notably, the TGN serves not only as a transport hub for NLRP3 but also as a critical site where these PTMs occur, particularly involving processes like palmitoylation and acetylation of NLRP3. Among these, the regulation of palmitoylation is notably intricate: zinc finger DHHC-type palmitoyl transferase family member ZDHHC7 catalyzes palmitoylation at the NLRP3 Cys126 residue, thereby promoting its localization and activation within the dTGN [133]. In contrast, palmitoylation at the Cys841 site mediated by ZDHHC12 exerts a negative feedback effect by inhibiting inflammatory signal transduction [134]. Additionally, TLR signaling and fatty acid synthase (FASN) can induce palmitoylation at the NLRP3 Cys898 site, further enhancing its interaction with dTGN [135]. Acetylation also plays a regulatory role: lysine acetyltransferase 5 (KAT5) catalyzes the acetylation of the Lys24 residue of NLRP3, which not only increases the formation of dTGN but also promotes the aggregation of NLRP3 within dTGN [136]. These findings collectively indicate that the TGN structurally remodels and functionally regulates NLRP3 through dynamic PTMs.

### The regulation of NLRP3 inflammasome by endosomes

Endosomes, serve as the central structure for sorting and transporting substances within cells, are categorized into early endosomes, late endosomes, and recycling endosomes based on their functional characteristics. Early endosomes internalize extracellular substances via endocytosis and perform initial sorting. Subsequently, these materials are transported to lysosomes through late endosomes for participation in autophagy and degradation processes. Recycling endosomes, on the other hand, are involved in the retrograde transport of materials within the cell [137, 138]. Notably, endosomes serve as critical platforms for intracellular signal transduction and play a central role in NLRP3 inflammasome activation. By integrating multiple functions, including recognition of membrane receptor signals, trafficking of endocytosed materials, and maintenance of membrane architecture and microenvironmental homeostasis, endosomes coordinate upstream stimuli that drive inflammatory activation.

#### TLR-endosome signaling and NLRP3 priming

Endosomes serve as essential compartments where TLRs recognize nucleic acids and initiate NLRP3 inflammasome activation. Upon recognition of RNA or DNA ligands, on one hand, TLRs upregulate NLRP3 expression at transcriptional and post-transcriptional levels via activation of the NF-κB and interferon regulatory factor (IRF) pathways [139–141]. On the other hand, TLRs directly facilitate NLRP3 activation through distinct signaling axes. For example, TLR7/8 activates the NLRP3 inflammasome via the hypoxia-inducible factor-1α (HIF-1α)-xanthine oxidase (XOD)-ROS axis [142]. The synergistic interaction between TLR8 and C-X-C motif chemokine 4 (CXCL4) targets the TBK1/inhibitor of NF-κB kinase ε (IKKε)-IRF5 pathway to promote K^+^ efflux, thereby amplifying the inflammatory cascade [143]. Downstream of TLR9, cyclic diadenylate (c-di-AMP), synthesized by adenylate cyclase 7 (ADCY7), has been shown to be critical for NLRP3 activation [144]. Inhibition of TLR9 significantly attenuates the NLRP3-mediated inflammatory response, further underscoring the functional relevance of this pathway [145]. It is noteworthy that endosomal TLR9 plays a critical role in fine-tuning the signaling intensity of non-canonical inflammatory pathways through negative regulation of the IFNβ-caspase-11 axis [146]. Notably, mtDNA has been demonstrated to drive central nervous system inflammation through TLR9, suggesting that crosstalk between mitochondria and endosomes may constitute a crucial node in the activation of central NLRP3 inflammasomes [147].

Beyond the TLR signaling pathway, endosomes are also central to the initiation of non-canonical inflammasome activation. Early endosomes serve as pivotal platforms for this process. Specifically, arachidonate-12-lipoxygenase (ALOX12)- mediated lipid peroxidation facilitates the translocation of LPS from early endosomes into the cytoplasm, thereby activating caspase-11 [148]. Additionally, outer membrane vesicles (OMVs) derived from Gram-negative bacteria promote the recruitment of caspase-5 to the early endosomal membrane through sorting nexin 10 (SNX10), enabling LPS release into the cytosol and triggering caspase-5-dependent inflammatory signaling [149].

#### Endosome-mediated endocytosis drives NLRP3 localization and activation

Endosomes function as dynamic signaling platforms that promote the activation of the NLRP3 inflammasome by orchestrating the trafficking and subcellular localization of inflammatory mediators. In the early endosomal compartment marked by ras-related protein 5 (Rab5), following endocytosis of the complement membrane attack complex (MAC), the zinc finger FYVE-type containing 21 (ZFYVE21) domain is translated and stably expressed in a Rab5-PI3P-dependent manner [150]. This subsequently activates the NF-κB-inducing kinase (NIK) signaling module, leading to stimulation of the non-canonical NF-κB pathway, synthesis of pro-IL-1β and NLRP3, and ultimately proteolytic processing and secretion of mature IL-1β [151]. Concurrently, ZFYVE21 interacts with Run domain protein as Beclin-1-interacting and cysteine-rich containing (Rubicon), a negative regulator of the late endosomal marker ras-related protein 7 (Rab7), and the E3 ubiquitin ligase ring finger protein 34 (RNF34), forming the ZFYVE21-Rubicon-RNF34 (ZRR) signaling complex. This complex enhances the activity of NLRP3 inflammasome by relieving the inhibitory binding of caspase-1 to its pseudosubstrate Flightless I (FliI) and degrading FliI, thereby expanding endosome-associated caspase-1 [152]. On the other hand, Rab7 drives the NLRP3 inflammatory response following the endocytosis of inflammatory stimuli such as genomic DNA [153]. Furthermore, endosomes can also facilitate the activation of the NLRP3 inflammasome by promoting the localization of proteins, such as NADPH oxidase 2 (NOX2) and calcitonin gene-related peptide (CGRP), into endosomes. This further enhances the role of endosomes as a “signal integration platform” for the NLRP3 inflammatory response [154–156].

#### Endosomal membrane integrity modulates NLRP3 inflammasome activation

The structural integrity of the endosomal membrane is a critical determinant of its functionality and inflammatory activity. Physical damage induced by factors such as silicon dioxide particles (SPs) and high-density polyethylene (HDPE) can compromise the integrity of the intracellular membrane, leading to the aberrant release of cathepsin (CTS) and subsequent activation of the NLRP3 inflammasome [157, 158]. Conversely, annexin A2 (ANXA2) repairs damaged endosomal membranes via a Ca^2+^/H^+^-dependent mechanism, thereby significantly inhibiting NLRP3 activation [158]. This suggests that stabilizing endosomal membrane integrity may represent a novel therapeutic strategy for modulating inflammatory responses. Notably, the process of endosomal acidification further influences NLRP3 inflammasome activation by regulating ion gradients across the membrane. This phenomenon is associated with the localization of transmembrane protein 176B (TMEM176B) on the endosomal membrane [159, 160]. These findings collectively demonstrate that dynamic alterations in the endosomal microenvironment exert precise regulatory effects on inflammatory signaling pathways.

### The regulation of NLRP3 inflammasome by lysosome

Lysosomes, serving as the primary degradation centers within cells, encapsulate hydrolases via their single-layer membrane structure to break down macromolecular substances, damaged organelles, and abnormal protein aggregates transported by phagosomes. This process is intricately linked to autophagy [161]. In the classical autophagy pathway, autophagosomes first engulf the target substances, subsequently fuse with lysosomes to form autophagolysosomes, and ultimately rely on lysosomal enzymes to complete the catabolic degradation of these substances [162, 163]. Notably, the regulation of autophagy and the NLRP3 inflammasome exhibits a bidirectional relationship: moderate autophagy flux typically exerts anti-inflammatory effects, whereas impaired autophagy flux exacerbates the activation of the NLRP3 inflammasome [164, 165].

#### Autophagy dysfunction drives NLRP3 inflammasome activation

Lysosomal-associated membrane protein 1 (LAMP1), LAMP2, and transcription factor EB (TFEB) are key regulators of lysosomal autophagy, and their functional states directly influence the magnitude of the NLRP3 inflammatory response. Evidence indicates that suppression of LAMP1 transcription increases phagolysosomal membrane permeability (PMP), impairs autophagy, and activates the LAMP1–NLRP3 axis, thereby exacerbating inflammation [166, 167]. Similarly, deficiency in LAMP2A impedes chaperone-mediated autophagy (CMA), leading to impaired degradation of NLRP3 [168]. TFEB plays a pivotal role in modulating NLRP3-driven inflammation. Knockdown of TFEB in microglia reduces autophagic clearance of NLRP3, thereby intensifying neuroinflammatory responses [169]. Furthermore, its upstream regulator Poly (ADP-ribose) Polymerase 1 (PARP1) inhibits autophagy and blocks NLRP3 degradation by promoting ADP-ribosylation of TFEB and phosphorylation at Ser211 [170]. Conversely, activation of TFEB enhances autophagic flux and facilitates NLRP3 clearance by increasing the LC3-II/I ratio and upregulating LAMP1 expression [169]. Thus, modulation of the PARP1-TFEB-NLRP3 pathway holds neuroprotective potential and may serve as a viable target for therapeutic intervention. Moreover, proteins such as recombinant human autophagy effector protein Beclin-1, syntaxin 17 (STX17), and vesicle-associated membrane protein 8 (VAMP8) play critical roles in the fusion of autophagosomes and lysosomes. Functional defects in these proteins result in the accumulation of undegraded substances, thereby directly activating the NLRP3 inflammasome response [171, 172]. Conversely, enhancing autophagy can effectively suppress the occurrence of the NLRP3 inflammasome response [173]. In summary, enhancing autophagy exerts an overall inhibitory effect on the NLRP3-mediated inflammatory response.

#### Lysosomal membrane damage triggers the NLRP3 inflammasome response

Lysosomal membrane integrity is critical in regulating NLRP3 inflammasome activation. Upon phagocytosis of exogenous particles (e.g., silica crystals, asbestos) or endogenous deposits (e.g., cholesterol crystals, Aβ), lysosomes may undergo rupture, leading to the release of CTSB. CTSB subsequently interacts with NLRP3 to trigger inflammasome assembly, promoting cellular inflammation and pyroptosis [174–180]. Notably, CTSB not only directly interacts with NLRP3 but also activates caspase-11, which enhances the fusion of pathogen-containing vesicles with lysosomes through modulation of the RhoA-cofilin signaling axis, thus creating favorable conditions for NLRP3 activation [181, 182]. Lysosomal membrane destabilization can also arise from endogenous mechanisms, such as activation of the transforming growth factor-β-activated kinase 1 (TAK1) signaling pathway or aberrant phospholipid metabolism, both of which contribute to CTSB leakage and ion imbalance, thereby synergistically driving NLRP3 activation [183–187]. Furthermore, upon lysosomal membrane rupture, galectin-8 (Gal8) rapidly recruits caspase-4 to the surface of intracellular bacteria to detect cytosolic LPS, leading to GSDMD-mediated pyroptosis [188]. Importantly, lysosomes contribute to non-canonical inflammasome pathways through galectin-3 (Gal3) as well. Specifically, Gal3 and LPS co-accumulate in early endosomes and are subsequently co-transported to lysosomes. This process promotes LPS internalization through both receptor of advanced glycation endproducts (RAGE)-dependent and RAGE-independent mechanisms, thereby amplifying the inflammatory response mediated by the caspase-4/11-GSDMD pyroptotic pathway [189]. Collectively, these findings indicate that lysosomes function not only as key intracellular hubs for inflammation regulation but may also participate in the formation of neuroinflammatory networks by mediating intercellular cargo transport and initiating non-canonical inflammatory signaling cascades.

### The regulation of NLRP3 inflammasome by centrosome

Centrosomes, which function as membraneless organelles, mediate microtubule nucleation via their paired structures and the surrounding pericentriolar material (PCM), thereby playing a pivotal role in spindle assembly and cell division. The PCM facilitates microtubule growth by anchoring the γ-tubulin ring complex (γ-TuRC) and sustains the dynamic equilibrium of the intracellular microtubule network [190]. Emerging evidence has revealed its involvement in the fine-tuned regulation of the NLRP3 inflammasome. Mechanistically, the centrosome modulates NLRP3 activation not only through dynamic modifications and protein-protein interactions of its associated components but also by serving as a microtubule-anchoring site that guides the spatial organization of inflammatory signaling molecules [191]. These findings collectively establish the centrosome as a pivotal regulatory node in innate immune responses.

#### Regulation of the NLRP3 inflammasome by centrosome-related proteins

Centrosome-related proteins play a critical role in modulating the activity of the NLRP3 inflammasome through dynamic modification and interaction networks. Notably, NEK7 serves dual functions: it acts as a key regulator for centriole replication and PCM assembly, while also promoting NLRP3 activation by disrupting its self-inhibitory cage structure or dissociating inactive NLRP3 polymers to form the active NEK7–NLRP3 dimer, thereby directly driving the activation of the NLRP3 inflammasome [192, 193]. However, NEK7 is not essential for NLRP3 oligomerization, which accounts for its independent activation in certain contexts [192, 194]. Furthermore, polo-like kinase 4 (PLK4)-mediated phosphorylation of NEK7 induces negative feedback inhibition by weakening the NEK7–NLRP3 interaction [195]. This mechanism involves surfactant associated protein A2 (SPATA2) recruiting the deubiquitinating enzyme cylindromatosis (CYLD) to the centrosome, stabilizing PLK4 activity and enhancing its interaction efficiency with NEK7, thus indirectly regulating NLRP3 signaling intensity [195]. Notably, abnormalities in the number, structure, or function of centrosomes, referred to as centrosome disorder, can activate the NLRP3 inflammasome through specific molecular mechanisms. For instance, pericentrin (PCNT) and PCM1, key constituents of PCM, can induce pyroptosis via the NLRP3-GSDMD pathway upon stimulation by activators such as nigericin. This elucidates the direct link between centrosomes and inflammatory responses [191].

#### Microtubule-organizing center (MTOC) mediates the localization and activation of NLRP3

The centrosome, as a core component of the MTOC, regulates the subcellular localization and activation threshold of NLRP3 via the microtubule network. Upon binding to NLRP3, microtubule affinity-regulating kinase 4 (MARK4) facilitates its migration to the MTOC, thereby forming “NLRP3 spots.” Disruption of the MARK4–NLRP3 interaction impairs microtubule-dependent localization and restricts inflammasome activation [196]. Additionally, polo-like kinase 1 (PLK1) orchestrates MTOC functionality through a dual mechanism. During interphase, PLK1 promotes the recruitment of γ-tubulin to the PCM, ensuring the maintenance of MTOC maturity. Notably, PLK1 not only contributes to MTOC formation but also regulates the anchoring of NLRP3 to MTOC and mediates the release of IL-1β. Inhibition of PLK1 disrupts microtubule dynamics and significantly attenuates NLRP3 signaling [190]. Collectively, these findings underscore the critical role of MTOC in integrating cytoskeletal homeostasis with inflammatory responses.

Furthermore, as the central platform for lipid storage and metabolic regulation, an imbalance between the formation and degradation of lipid droplets has emerged as a critical factor in initiating inflammatory responses. Under high-fat conditions, fatty acid binding protein (FABP) not only promotes the formation of lipid droplets but also mediates the transmission of pro-inflammatory signals to the NLRP3 inflammasome, thereby triggering inflammation [197]. Concurrently, tripartite motif-containing 59 (TRIM59) induces ubiquitin-mediated degradation of the lipolysis co-activator abhydrolase domain containing 5 (ABHD5). The resulting deficiency of ABHD5 suppresses lipid breakdown, leading to lipid droplet accumulation, activation of the NLRP3 inflammasome [198]. Notably, cholesterol crystals formed within lipid droplets have been shown to directly promote NLRP3 inflammasome activation. However, downregulation of key lipid synthesis enzymes, such as FASN, can reduce intracellular levels of cholesterol crystals and triglycerides, thereby suppressing NLRP3 activation and mitigating hepatic inflammation [199, 200]. The impaired function of the liver X receptor β (LXRβ)/ATP-binding cassette transporter A1 (ABCA1) pathway leads to intracellular lipid droplet accumulation and subsequent activation of NLRP3 inflammasome, primarily due to reduced ABCA1-mediated cholesterol efflux capacity [201].

**Fig. 3:**
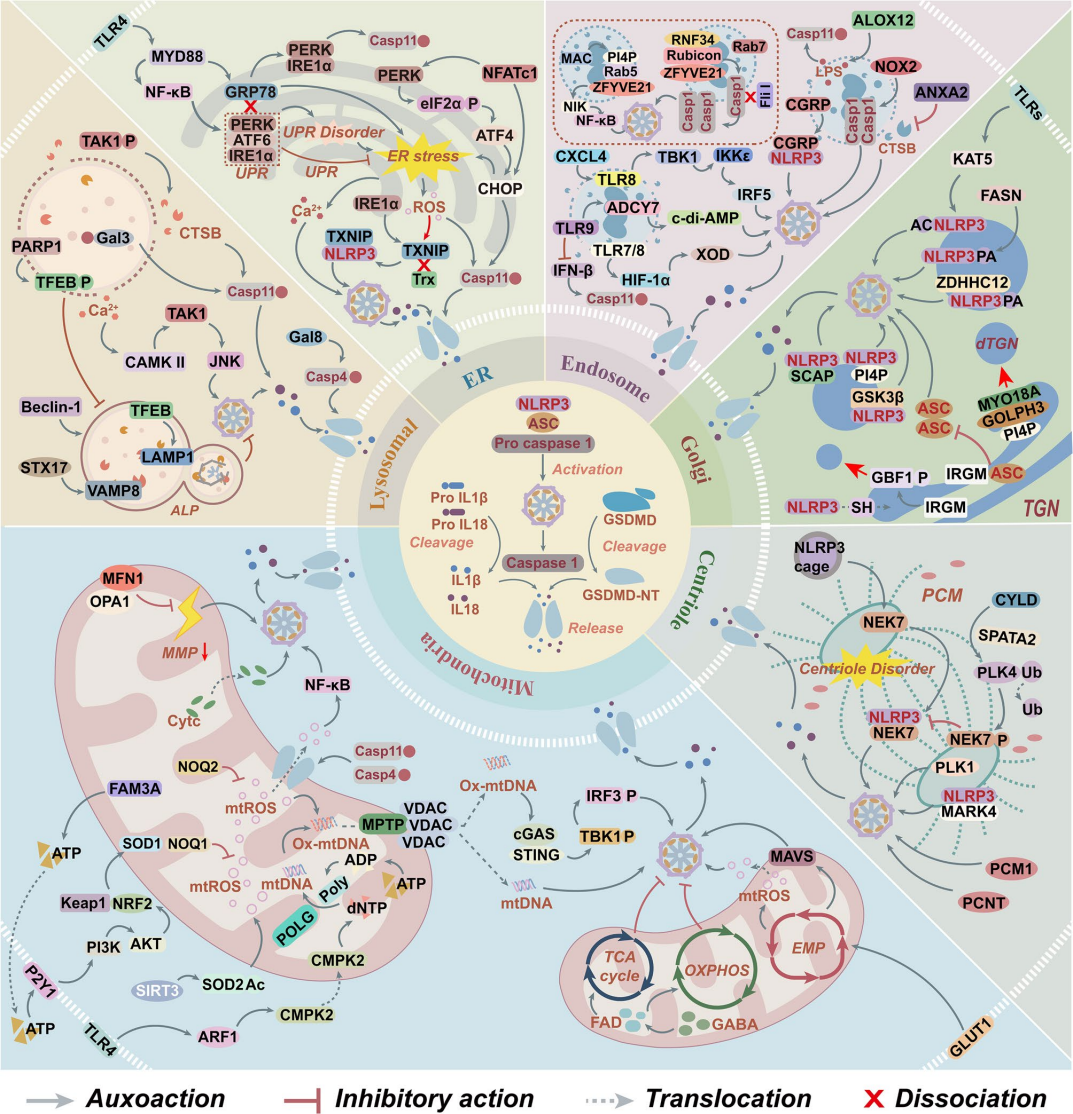
The mechanism by which organelle stress mediates the NLRP3 inflammasome activation. The mechanism of NLRP3 inflammasome activation is intricately linked to the precise regulation of organelle stress. Specifically, mitochondrial stress triggers the cGAS-STING and MAPK pathways via mtROS and mtDNA, while simultaneously modulating metabolic pathways such as the TCA cycle and OXPHOS to facilitate inflammasome activation. ERS promotes NLRP3 assembly through Ca^2+^ release or the TXNIP signaling pathway. During golgi remodeling, the MYO18A-PI4P complex induces the formation of the dTGN, which enhances NLRP3 activity upon recruitment through PTMs such as palmitoylation and acetylation. Lysosomes mediate NLRP3 activation by releasing CTSB and activating it along the Ca^2+^-CAMKII-TAK1-JNK axis; however, autophagy mediated by beclin-1 or STX17–VAMP8 complex inhibits this process. In endosomes, NOX2 and CGRP promote caspase-1 oligomerization to activate inflammasomes; whereas ANXA2 attenuates the release of CTSB, inhibiting its activation. Furthermore, NEK7 and MARK4 enhance NLRP3 activation through microtubule localization and centriole-associated proteins, but phosphorylation of NEK7 disrupts their interaction, thereby exerting negative regulation. Abbreviations: ADCY7, adenylate cyclase 7; ALOX12, arachidonate-12-lipoxygenase; ANXA2, annexin A2; ASC, apoptosis-associated speck-like protein containing card; ARF1, ADP-ribosylation factor 1; ATF, activating transcription factor; AKT, protein kinase B; CAMK II, Calcium/calmodulin-dependent protein kinase II; Casp/caspase, cysteine aspartate-specific protease; cGAS, cyclic GMP-AMP synthase; CGRP, calcitonin gene-related peptide; CHOP, C/EBP homologous protein; CTSB, cathepsin B; CMPK2, Cytidine/uridine monophosphate kinase 2; CXCL4, C-X-C motif chemokine 4; CYLD, Cylindromatosis; Cytc, cytochrome c; dTGN, dispersed trans-golgi network; eIF2α, eukaryotic initiation factor 2α; EMP, embden-meyerhof-Parnas; ER, endoplasmic reticulum; FAD, flavin adenine dinucleotide; FAM3A, family with sequence similarity 3-member A; FASN, TLR signaling and fatty acid synthase; FliI, flightless I; GABA, gamma-aminobutyric acid; Gal, galectin; GBF1, golgi-specific brefeldin A-resistance factor 1; GLUT1, glucose transporter 1; GOLPH3, golgi phosphoprotein 3; GRP78, glucose-regulated protein 78; GSDMD, gasdermin D; GSDMD-NT, GSDMD N-terminal domain; GSK-3β, glycogen synthase kinase 3β; HIF-1α, hypoxia-inducible factor-1α; IFNβ, interferon-β; IKKε, inhibitor of NF-kB kinase ε; IRE1α, inositol-requiring enzyme 1α; IRF, interferon regulatory factor; IRGM, immunity-related GTPase M; JNK, c-Jun N-terminal kinase; KAT5, lysine acetyltransferase 5; Keap1, kelch-like ECH-associated protein 1; LAMP1, lysosomal-associated membrane protein 1; LPS, Lipopolysaccharide; MAC, complement membrane attack complex; MAPK, mitogen-activated protein kinase; MARK4, microtubule affinity-regulating kinase 4; MAVS, mitochondrial antiviral signaling protein; MFN1, mitofusin 1; MMP, mitochondrial membrane potential; MPTP, mitochondrial permeability transition pore; mtDNA, mitochondrial DNA; mtROS, mitochondrial reactive oxygen species; MYD88, myeloid differentiation primary response protein 88; MYO18A, myosin XVIIIA; NEK7, NIMA-related kinase 7; NFATc1, nuclear factor of activated T cells cytoplasmic 1;NF-κB, nuclear factor kappa B; NIK, NF-κB-inducing kinase; NLRP3, the NOD-like receptor family pyrin domain-containing 3; NOX2, NADPH oxidase 2; NRF2, nuclear factor erythroid 2-related factor 2; OPA1, mitochondrial dynamin-like GTPase; OXPHOS, oxidative phosphorylation; ox-mtDNA, oxidized-mitochondrial DNA; OXPHOS, oxidative phosphorylation; PARP1, poly (ADP-ribose) polymerase 1; PCM, pericentriolar material; PCNT, Pericentrin; PERK, protein kinase R-like er kinase; PI3K, phosphatidylinositol 3-kinase; PI4P, phosphatidylinositol 4-phosphate; PLK, polo-like kinase; PTMs, post-translational modifications; Rab, ras-related protein; RNF34, ring finger protein 34; Rubicon, run domain protein as beclin-1-interacting and cysteine-rich containing; SCAP, SREBP cleavage-activating protein; SOD, superoxide dismutase; STING, stimulator of interferon genes; STX17, syntaxin 17; SPATA2, surfactant-associated protein A2; ROS, reactive oxygen species; TAK1, transforming growth factor-β-activated kinase 1; TBK1, tank binding kinase 1; TCA, tricarboxylic acid; TFEB, transcription factor EB; TGN, trans-golgi network; TLR, toll-like receptor; TRX, Thioredoxin; TXNIP, thioredoxin-interacting protein; UPR, unfolded protein response; VAMP8, vesicle-associated membrane protein 8; VDAC, voltage-dependent anion channel; XOD, xanthine oxidase; ZDHHC12, zinc finger DHHC-type palmitoyl transferase 12; ZFYVE21, zinc finger FYVE-type containing 21

To conclude, the activation mechanism of the NLRP3 inflammasome is intricately linked to the organelle stress network. Its core regulatory factors can be categorized into three primary aspects: First, the disruption of organelle structure and abnormal leakage of their contents can trigger the inflammatory cascade reaction; Second, the endosomal system and centrosome-MTOC microtubule network mediate the targeted localization of NLRP3 to lysosomes, endosomes, and TGN; Third, the TGN serves as a platform for PTMs such as phosphorylation and ubiquitination, dynamically regulating the activation of the NLRP3 inflammasome through structural remodeling of NLRP3. Lysosomes exhibit bidirectional regulatory functions: membrane rupture releases pro-inflammatory factors to activate NLRP3, while the autophagy pathway clears abnormal components to inhibit its activation. Based on these insights, multi-stage interventions can be implemented during the activation process (Fig. 3). Specifically, blocking endosomal sorting or enhancing lysosomal autophagy in the early stage, inhibiting microtubule transport or intervening in TGN-mediated modifications in the middle stage, and stabilizing membrane structures or modulating ion channels in the later stage. This stratified approach provides a novel and precise direction for targeting the NLRP3 inflammasome activation process.

## The regulatory mechanism of organelle interaction and NLRP3 inflammasome

Organelles, including mitochondria, ER, Golgi apparatus, endosome, and lysosome, maintain functional independence via specialized membrane structures. Simultaneously, they rely on membrane contact sites (MCSs) to facilitate material exchange and inter-organelle signal transduction [202]. For instance, mitochondria function as “energy factories” by providing energy support to other organelles, while lysosomes sustain cellular homeostasis through waste elimination. Nevertheless, the disruption of the interaction network among organelles may lead to the aberrant activation of the NLRP3 inflammasome, which serves as a critical inducer of inflammatory responses. Although the NLRP3 inflammatory response has been recognized as an essential indicator of organelle interaction dysregulation, its precise regulatory mechanism remains to be systematically elucidated. A comprehensive investigation into the dynamic principles governing the organelle interaction network during NLRP3 activation can not only uncover the molecular mechanisms underlying inflammation regulation but also offer novel strategies for targeted intervention in inflammatory diseases.

### Endosome-lysosome interaction

Endolysosomes are primary lysosomes formed through the fusion of endosomes and lysosomes, containing inactive hydrolases that potentially suppress the NLRP3 inflammasome response. Studies have demonstrated that nanoparticle-associated molecular patterns (NAMPs) induce abnormal activation of the NLRP3 inflammasome through disrupting endosome stability, inhibiting endosome-lysosome fusion, and impairing autophagy. These findings suggest that endolysosomal dysfunction may directly trigger inflammatory signaling pathways [203]. Moreover, inhibiting endolysosomal acidification or blocking the excessive activation of the key enzyme CTSB can effectively alleviate the NLRP3 inflammatory cascade reaction, offering a novel strategy for targeting lysosomal homeostasis in the treatment of inflammatory diseases [204].

### Mitochondria-lysosome interaction

Studies have demonstrated that the pathological accumulation of autophagosomes and selective autophagy defects in organelles such as mitochondria, caused by lysosomal dysfunction, can promote the NLRP3 inflammatory response by reducing autophagy flux [205]. Mitochondrial autophagy serves as a critical quality control mechanism, selectively isolating damaged mitochondria into autophagosomes for subsequent lysosomal degradation [206]. However, external stimuli, including ethanol, copper, and silver nanoparticles, can interfere with this process, resulting in the accumulation of mitochondrial damage and the release of mtROS and mtDNA [207–209]. These abnormal products not only directly activate the NLRP3 inflammasome but also further impair lysosomal function, thereby establishing a vicious cycle of autophagy deficiency and inflammatory amplification [210]. Notably, MitoTEMPO, which targets the clearance of mtROS, can effectively block these inflammatory cascade reactions, indicating that modulating the mitochondrial autophagy pathway may represent a promising strategy for intervening in NLRP3 activation [211].

PTEN-induced kinase 1 (PINK1) - E3 ubiquitin ligase Parkin, along with unc-51-like autophagy activating kinase 1 (ULK1) signaling pathway is the core regulatory mechanism of mitochondrial autophagy [212]. A variety of proteins dynamically modulate the activity of the NLRP3 inflammasome by influencing mitochondrial autophagy. For instance, signal transducer and activator of transcription 3 (STAT3) and fructose-bisphosphate aldolase A (ALDOA) inhibit the PINK1-Parkin pathway, leading to mitochondrial autophagy deficiency and subsequently driving the activation of the NLRP3 inflammasome. In this process, NF-κB and cGAS-STING may serve as secondary effector factors following pathway inhibition, further amplifying the inflammatory signal [208, 213–215]. Conversely, triggering receptor expressed on myeloid cells 2 (TREM2) and NRF2 enhance mitochondrial autophagy efficiency by activating the PINK1-Parkin pathway, thereby suppressing NLRP3 inflammasome activation [216–218]. Additionally, AMP-activated protein kinase (AMPK), acting as a downstream effector of this pathway, promotes mitochondrial autophagy via its phosphorylation, reducing the NLRP3 inflammatory response. These findings suggest that targeting the mitochondrial autophagy regulatory network offers potential therapeutic strategies for inflammatory diseases [219].

In addition to these core pathways, other signaling axes contribute to the regulation of mitophagy and influence NLRP3 inflammasome activity. For instance, PPARγ coactivator-1α (PGC-1α) promotes mitochondrial autophagy by activating ULK1, thereby suppressing the activation of the NLRP3 inflammasome [220]. The protein kinase C (PKC)-NRF2 pathway directly inhibits the NLRP3 inflammatory response by synergistically enhancing mitochondrial autophagy capacity [221]. The silent information regulator 2 homolog 1 (SIRT1)-Rab7 axis mediates mitochondrial autophagy via a late endosomal-dependent mechanism, attenuating STING signal transduction and blocking NLRP3 activation [222]. In summary, oxidative stress or external stimuli lead to the accumulation of damaged mitochondria and the release of mtROS or mtDNA by interfering with lysosomal function or the core pathway of mitochondrial autophagy, establishing a vicious cycle of “autophagy deficiency-inflammatory amplification” that ultimately drives the activation of the NLRP3 inflammasome. This mechanism highlights the pivotal role of the bidirectional interaction between autophagy imbalance and the inflammatory signaling cascade in NLRP3-related pathological processes.

### Mitochondria-ER interaction

Mitochondria and ER interact closely via a dynamic contact structure known as mitochondria-associated membranes (MAMs). These interactions maintain an optimal proximity between the two organelles and regulate critical biological processes, including signal transduction, redox reactions, and mitochondrial dynamics [223–225]. Research has demonstrated that MAM-dependent calcium homeostasis regulation serves as one of the critical triggering mechanisms for the NLRP3 inflammasome-mediated inflammatory response, with its functional integrity being essential for inhibiting the inflammatory cascade [226]. ERS or mitochondrial dysfunction can impair the structural and functional integrity of MAMs, leading to calcium homeostasis imbalance and the release of mitochondrial DAMPs, and subsequent activation of the NLRP3 inflammasome, thereby triggering an inflammatory response [227, 228]. These findings underscore the importance of MAMs integrity as a pivotal molecular node in regulating cellular homeostasis and inflammatory balance.

Molecular complexes at MAMs, including the inositol 1,4,5-trisphosphate receptor (IP3R)-glucose-regulated protein 75 (GRP75)-voltage-dependent anion channel 1 (VDAC1)-mitochondrial calcium uniporter (MCU), vesicle-associated membrane protein-associated protein B (VAPB), and protein tyrosine phosphatase-interacting protein 51 (PTPIP51), mediate calcium transport from ER to mitochondria [110, 229, 230]. Impairment in the structure or function of MAMs can result in dysfunction of these complexes, leading to excessive Ca^2+^ influx into mitochondria. This causes mitochondrial calcium overload and disrupts intracellular calcium homeostasis [7]. Furthermore, it triggers a Ca^2+^-dependent organelle stress response and affects ATP production, ultimately activating the NLRP3 inflammasome [231].

Furthermore, multiple protein signaling pathways regulate the activation of the NLRP3 inflammasome in a MAMs-dependent manner. On one hand, eATP enhances downstream signaling via pathways such as the P2X7R and the ERS-associated transcription factor CHOP, thereby exacerbating structural damage to MAMs and amplifying the NLRP3 inflammatory response [232, 233]. On the other hand, under the stimulation of homocysteine (Hcy), molybdenum (Mo), or other factors, damaged MAMs activate signaling pathways including TXNIP, AMPK, NF-κB, and cGAS-STING, driving the assembly and activation of the NLRP3 inflammasome [10, 234–237]. These findings suggest that MAMs serve not only as a core platform for calcium homeostasis regulation but also as a key hub for integrating signals from multiple pathways. The functional integrity of MAMs is critical for suppressing abnormal inflammatory responses. Targeting MAMs-related proteins or downstream signaling nodes may offer potential therapeutic strategies for modulating NLRP3 activation.

### ER-lysosomes interaction

The interaction between the ER and lysosomes is pivotal in maintaining cellular homeostasis. Misfolded proteins are cleared via lysosomal degradation, while signaling pathways such as cGAS-STING transmit signals through the ER-lysosome interface [238]. Research has demonstrated that impaired ER-lysosome interactions may trigger the NLRP3 inflammasome-mediated inflammatory response. Specifically, cytosolic double-stranded DNA (dsDNA) is recognized by cGAS, which activates STING and promotes its translocation from the ER to lysosomes. This process induces lysosomal membrane permeabilization (LMP), leading to lysosome-dependent cell death (LCD) and subsequent activation of the NLRP3 inflammasome via cytoplasmic K^+^ efflux [184, 238, 239]. These findings indicate that ER-lysosome dysfunction, characterized by lysosomal damage and ion homeostasis disruption, represents a critical mechanism underlying the NLRP3 inflammatory response.

It is noteworthy that cholesterol regulates the activation of the NLRP3 inflammasome via the ER-lysosome pathway [205]. Specifically, cholesterol is transported through the niemann-pick type C1 protein (NPC1)-ABCA1/ATP-binding cassette transporter G (ABCG1) system, which induces the upregulation of protein kinase A (PKA) and IP3R protein expressions in the ER, thereby promoting the release of Ca^2+^. Subsequently, it drives the phosphorylation of calcium/calmodulin-dependent protein kinase II (CaMKII) and c-Jun N-terminal kinase 1 (JNK-1), further promoting NLRP3 deubiquitination mediated by the BRCA1-associated ring domain protein 3 (BRCC3)/NLRP3 complex, and ultimately driving the activation of the NLRP3 inflammasome [240]. These studies elucidate the molecular mechanism by which abnormal cholesterol metabolism regulates inflammasome activation through ER-lysosomal interactions and calcium signaling cascade reactions, offering new insights for the intervention of metabolism-related inflammatory diseases.

### ER-Golgi interaction

The ER-Golgi interaction plays a critical role not only in fundamental processes such as protein synthesis, modification, and sorting but also in modulating inflammatory responses via inter-organelle communication. Research has demonstrated that the ER and Golgi apparatus can collaboratively facilitate the activation of the NLRP3 inflammasome via sterol regulatory element-binding protein 2 (SREBP2) and SREBP cleavage-activating protein (SCAP)-mediated signaling pathway. The underlying mechanism involves the formation of a complex between NLRP3 and SCAP-SREBP2 in the ER. This complex is subsequently transported from the ER to the Golgi apparatus, thereby facilitating the assembly and activation of the NLRP3 inflammasome [241, 242]. This process elucidates a novel mechanism by which ER-Golgi interactions regulate inflammatory signaling through membrane trafficking and dynamic reorganization of protein complexes.

### ER-endosome interaction

Research has demonstrated that eukaryotic cells facilitate communication via vesicle-mediated transport among membranous organelles. Among these, endosomes, as a critical membranous organelle, can drive the NLRP3 inflammasome response through their dynamic membrane systems. Notably, the abnormal accumulation of PI4P within endosomes serves as the central mechanism underlying NLRP3 recruitment and activation. Furthermore, the endoplasmic reticulum-endosome membrane contact sites (EECSs) and endosome-to-trans-Golgi network trafficking (ETT) pathways play pivotal roles in regulating NLRP3 inflammasome activation [243]. Specifically, when NLRP3 activators such as nigericin induce the accumulation of PI4P in endosomes and subsequently trigger endosomal trafficking dysfunction, this promotes the localization of NLRP3 within endosomes and activates inflammasomes. Reducing the level of PI4P in endosomes can markedly diminish the co-localization of NLRP3 with endosomes, thereby attenuating the inflammatory response [243–245]. This process elucidates the molecular pathway by which dynamic imbalances in endosomal membrane contact sites regulate NLRP3 activation through PI4P-dependent membrane transport abnormalities.

In addition, disrupted interactions between lipid droplets and various organelles can collectively trigger the NLRP3 inflammatory response through multiple signaling pathways. Specifically, lipid droplet accumulation establishes a positive feedback loop with ERS, leading to the upregulation of pro-inflammatory mediators such as NLRP3 [246]. This process also impairs mitochondrial function and suppresses PINK1/Parkin-mediated mitophagy, thereby exacerbating lipid metabolic dysfunction and promoting NLRP3 inflammasome activation [247]. Notably, palmitic acid has been shown to inhibit autophagy and lysosomal biogenesis, resulting in impaired lipid droplet degradation and subsequent autophagy-dependent activation of the NLRP3 inflammasome [248]. Furthermore, upon binding of oxidized low-density lipoprotein (oxLDL) to the CD36 receptor, lipid droplet accumulation is promoted on one hand, while on the other, mtROS leakage, CTSB release, and potassium efflux are synergistically induced. These events converge to facilitate caspase-1 activation and the maturation and secretion of IL-1β via multiple signaling cascades [249]. Collectively, these mechanisms underscore the pivotal role of lipid droplets as a critical nexus linking metabolic regulation and inflammatory responses.

**Fig. 4:**
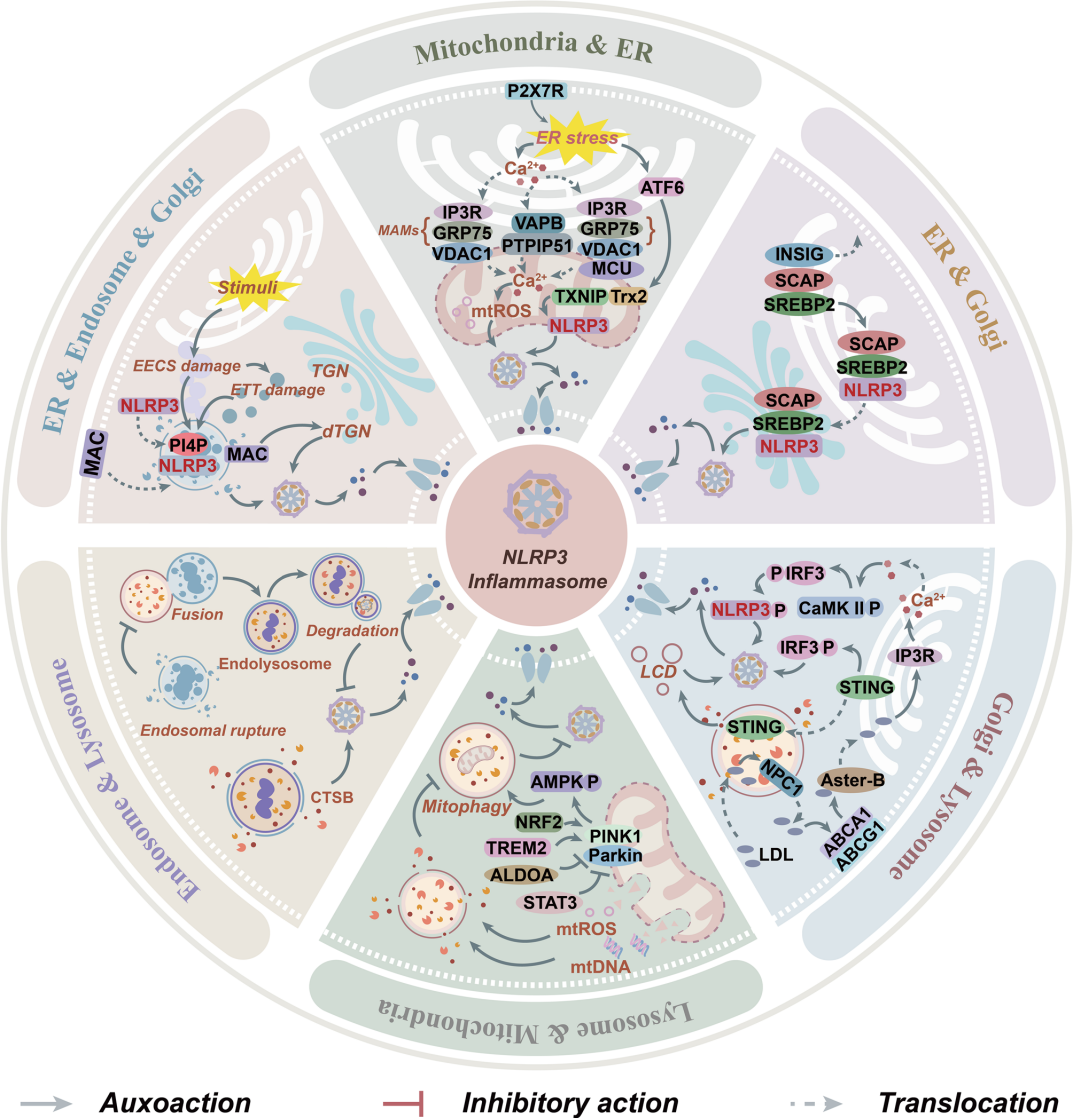
The role of organelle interactions in regulating the NLRP3 inflammatory response. Endolysosomes are capable of degrading the NLRP3 inflammasome; however, the rupture of endosomes and lysosomes inhibits this degradation process. Mitochondrial stress generates mtDNA and mtROS, which induce lysosomal damage and thereby inhibit mitophagy. In contrast, the PINK1-parkin pathway promotes mitophagy and reduces the activation of the NLRP3 inflammasome. ERS triggers Ca^2+^ release and mtROS production via the IP3R-GRP75–VDAC1 and VAPB-PTPIP51 complexes in MAMs, leading to the activation of the NLRP3 inflammasome. Damage to the ER induces EECS, while disruption of the ETT causes endosome rupture and disrupts the dTGN, further activating the NLRP3 inflammasome. SCAP-SREBP2 facilitates the translocation of NLRP3 from the ER to the golgi apparatus, thereby activating the NLRP3 inflammasome. The movement of LDL between lysosomes and the ER drives Ca^2+^ release, and the translocation of STING from the ER to lysosomes promotes LCD and activates the NLRP3 inflammasome. Abbreviations: ABCA1, ATP-binding cassette transporter A1; ABCG1, ATP-binding cassette transporter G1; ALDOA, fructose-bisphosphate aldolase A; AMPK, AMP-activated protein kinase; ATF6, activating transcription factor 6; CaMK II, Calcium/calmodulin-dependent protein kinase II; dTGN, dispersed trans-golgi network; EECS, endoplasmic reticulum-endosome membrane contact sites; ETT, endosome-to-trans-golgi network trafficking; GRP75, glucose-regulated protein 75; INSIG, insulin-induced gene; IP3R, inositol 1,4,5-trisphosphate receptor; IRF3, interferon regulatory factor 3; MAC, complement membrane attack complex; MCU, mitochondrial calcium uniporter; mtDNA, mitochondrial DNA; mtROS, mitochondrial reactive oxygen species; NLRP3, the NOD-like receptor family pyrin domain-containing 3; NPC1, niemann-pick type C1 protein; NRF2, nuclear factor erythroid 2-related factor 2; PINK1, PTEN-induced kinase 1; PI4P, phosphatidylinositol 4-phosphate; PTPIP51, protein tyrosine phosphatase-interacting protein 51; P2X7R, P2X purinergic receptor 7; SCAP, sterol regulatory element-binding protein cleavage-activating protein; SREBP2, sterol regulatory element-binding protein 2; STAT3, signal transducer and activator of transcription 3; STING, stimulator of interferon genes; TGN, trans-golgi network; TREM2, triggering receptor expressed on myeloid cells 2; Trx2, thioredoxin-2; TXNIP, thioredoxin-interacting protein; VAPB, vesicle-associated membrane protein-associated protein B; VDAC1, voltage-dependent anion channel 1

In conclusion, organelle interactions constitute a multi-level regulatory network in the inflammatory response mediated by the NLRP3 inflammasome via dynamic membrane contact, substance transport, and signal integration. The ER acts as a central regulatory hub: it modulates PI4P metabolism through interactions with endosomes via the EECS, thereby disrupting endosomal transport to the TGN and promoting the aggregation and activation of NLRP3. ER-Golgi interaction relies on the SP-SREBP2 complex to facilitate NLRP3 transport and assembly. ER-lysosome interaction triggers LMP and calcium release through cGAS-STING signaling and cholesterol transport pathways, while also cooperating with deubiquitination mechanisms to activate NLRP3. Moreover, the endolysosomal system directly activates NLRP3 via LMP and potassium efflux. Mitochondrial autophagy indirectly regulates inflammation by clearing damaged mitochondria and inhibiting ROS accumulation. The MAM, serving as a nexus for ER-mitochondria interaction, coordinates calcium signaling with ROS production to drive inflammasome activation (Fig. 4 and Table 1). These mechanisms suggest that targeting the organelle interaction network could provide precise intervention strategies for modulating the NLRP3-mediated inflammatory response.

**Table 1:** The regulatory mechanism of organelle interaction and NLRP3 inflammasome

Interaction Type	Target/ Pathway	Outcome on NLRP3	Reagents for model	Model	Disease	Reference
Endosome-lysosome	ROS-Ca^2+^	Activator	mRNA lipid nanoparticles			[203]
	CTSB-IL-1β	Activator	FhHDM-1	BMDMs	Helminth Infections	[204]
Mitochondria-lysosome	PINK-Parkin	Inhibitor	EtOH-LPS	Raw264.7 cells/A549 cells/C57BL/6 mice	Acute respiratory distress syndrome (ARDS)/Chronic obstructive pulmonary disease (COPD)	[207]
			CuSO_4_·5 H_2_O	BV2 cells/C57BL/6J mice	PD	[208]
			AgNPs	RAW264.7 cells/J774A0.1 cells/C57BL/6 J mice	Liver inflammation	[209]
			CIH	BV2 cells	Obstructive sleep apnea (OSA)	[212]
	TREM2- PINK-Parkin	Inhibitor		C57/BL6 mice	Postoperative cognitive dysfunction (POCD)	[216]
	NRF2- PINK-Parkin	Inhibitor	Alcohol	BV2/C57/BL6 mice	Chronic alcohol exposure	[217]
	SIRT3-Forkhead box protein O3a (FOXO3a)-Parkin	Inhibitor			Intervertebral disc degeneration (IVDD)	[218]
	PGC-1α-ULK1	Inhibitor			Acute ischemic stroke (AIS)	[220]
	PKC-NRF2	Inhibitor	tert‑butyl hydroperoxide		Osteoarthritis (OA)	[221]
	SIRT1-Rab7	Inhibitor	EX527	C57BL/6J mice/	Acute lung injury (ALI)	[222]
	ALDOA- AMPK	Activator	SLO/ATP/Nigericin/LPS	J774A0.1 cells/THP-1 cells/BMDMs/C57BL/6 J mice		[214]
	XBP1- cGAS-STING	Activator		BMDMs	Acute liver injury	[215]
Mitochondria-ER	IP3R- GRP75–VDAC1	Activator	High tidal volume (HTV)	MLE12 cells/C57BL/6 mice	Ventilator-induced lung injury (VILI)	[110]
			Mo/Cadmium (Cd)	Sheep hepatocytes	Hepatotoxicity	[234]
	VAPB-PTPIP51	Activator	Pseudomonas aeruginosa	S9 Cells/IB3-1 Cells		[230]
	Promyelocytic Leukemia Protein (PML)-P2X7R	Activator	LPS and Nigericin/LPS and Bz-ATP	B16–F10 melanoma cells/The LL/2 mouse Lewis lung carcinoma cell line/H460 cells/PBMCs/pRMØs/BMDMs	Tumor microenvironment (TME)	[232]
	CHOP-TXNIP	Activator		Primary podocytes/Primary TECs/Primary PTCs/Lamb2−/− mice/Txnip−/− mice/Chop−/− mice/	Nephrotic syndrome (NS)	[233]
	Nrf2-NQO1	Activator	Monosodium Iodoacetate (MIA)	OA rat	OA	[235]
	AMPK-mTOR	Activator		PBMCs	Hidradenitis suppurativa (HS)	[236]
	cGAS-STING	Activator	LPS	HK2 cells/C57BL/6J mice/STING^fl/fl^ mice	Acute kidney injury (AKI)	[237]
ER-lysosome	cGAS-STING	Activator	Ferric chloride	BV2 cells/C57BL/6 J mice	Cerebral venous sinus thrombosis (CVST)	[238]
			LPS/Pam3CSK4	BLaER1 cells/Primary human monocytes/HEK293T cells/		[184]
			LPS	NRCMs/H9c2 cells/C57/B6 mice	Sepsis/Sepsis-induced cardiomyopathy (SIC)	[239]
ER-Golgi	SCAP-SREBP2	Activator	ATP/Nigericin/LPS/MSU/Alum	BMDMs		[242]
ER-Endosome	EECS/ETT	Inhibitor	Nigericin/CL097	HeLa cells		[243]
Lipid droplet-ER	Gα12/Gα13/Vascular endothelial growth factor (VEGF)	Activator	High-fat diet (HFD)/Streptozotocin (STZ)/	C57/B6 mice	Diabetes	[246]
Lipid droplet-mitochondria	PINK-Parkin	Inhibitor	HFD	C57/B6 J mice	Nonalcoholic fatty liver disease (NAFLD)	[247]
Lipid droplet-lysosome	Ca^2+^-calcineurin-TFEB	Inhibitor	Palmitate (PA)/HFD	AML12 cells/Primary hepatocytes/C57BL/6 J mice/TFEB^f/f^ mice	NAFLD	[248]
Lipid droplet-lysosome	ROS-CD36	Activator	oxLDL	THP-1 cells	Vascular disease	[249]

## The role of organelle stress and NLRP3 inflammatory response in NDDs

NDDs, such as AD, PD, multiple sclerosis (MS), ALS, and Huntington’s disease (HD), are characterized by complex pathogenic mechanisms. Common pathological features include pathological protein aggregation, disrupted protein homeostasis, inflammation, and neuronal death [250]. While traditional research has primarily focused on the role of pathological proteins in driving these diseases, emerging evidence highlights that the abnormal activation of the NLRP3 inflammasome not only exacerbates NDD pathology but also serves as a pathological consequence, creating a vicious cycle that accelerates disease progression. Notably, NLRP3 inflammatory signals mediated by organelle interactions and their stress responses may play a pivotal role in the onset and progression of NDDs [251, 252]. Consequently, elucidating the regulatory mechanisms of organelle stress and its dynamic interaction network in modulating the NLRP3 inflammatory response could provide novel strategies for targeted interventions in NDDs.

### The organelle stress and NLRP3 inflammatory response regulation in AD

AD is an age-related NDD, Aβ deposition and neurofibrillary tangles (NFTs) represent not only core pathological hallmarks but also critical upstream events that induce organelle stress and trigger activation of the NLRP3 inflammasome [253]. Specifically, Aβ oligomers can directly interact with NLRP3, leading to inflammasome activation [254]. The subsequent organelle dysfunction, in conjunction with the NLRP3-mediated inflammatory cascade, constitutes a central mechanism that exacerbates neuronal loss and disease progression. During this process, microglia and astrocytes establish an inflammatory amplification network through extensive signaling crosstalk [255]. As primary sensors of pathological stimuli such as Aβ and tau, microglia initiate NLRP3 activation and shift toward the pro-inflammatory M1 phenotype [256]. Subsequently, microglia-derived factors, including IL-1β, promote the transition of astrocytes from the neuroprotective A2 state to the neurotoxic A1 phenotype [257–259]. This establishes a microglia-driven inflammatory axis that enhances astrocytic pro-inflammatory responses via NLRP3 activation, thereby contributing to a self-sustaining feedback loop that aggravates neurodegeneration [260, 261]. Notably, this inflammatory network is significantly modulated by key genetic risk factors associated with AD. For example, different isoforms of apolipoprotein E (APOE) exert opposing effects by regulating organelle stress responses. The pathogenic APOE4 isoform readily disrupts organelle homeostasis, facilitating NLRP3 activation [262], whereas the protective APOE2 isoform attenuates inflammation by reinforcing cellular protective mechanisms [263]. Similarly, mutations in the TREM2 gene, predominantly expressed in microglia, are strongly implicated in the NLRP3-dependent neuroinflammatory processes underlying AD [264].

#### Mitochondrial stress driving NLRP3 inflammatory response in AD

The pathogenesis of AD involves a self-perpetuating cycle between mitochondrial dysfunction and NLRP3 inflammasome activation. Traditionally, mitochondrial impairment is considered an early pathological event that precedes Aβ deposition and tau protein hyperphosphorylation [265]. This dysfunction contributes to Aβ production and tau phosphorylation through the activation of the TLR4-NF-κB signaling pathway, which in turn promotes NLRP3 inflammasome assembly and activation [266]. In this context, key genetic risk factors such as APOE and TREM2 play pivotal roles in regulating mitochondrial homeostasis. The APOE isoform enhances mitochondrial permeability transition pore (MPTP) opening by interacting with VDAC1, resulting in mitochondrial swelling and functional decline [267]. Furthermore, APOE suppresses FOXO3a-PINK1-mediated mitophagy, leading to the release of mtDNA and mtROS, thereby indirectly activating the NLRP3 inflammasome via the cGAS-STING pathway, thus amplifying neuroinflammatory responses [268–271]. In contrast, the APOE2 isoform exerts neuroprotective effects by binding to estrogen-related receptor α (ERRα), restoring MMP, and reducing oxidative stress [272]. Loss-of-function mutations or deficiency in TREM2 impair microglial energy metabolism and hinder the intercellular transfer of healthy mitochondria to neurons via tunneling nanotubes, exacerbating oxidative stress and facilitating NLRP3 activation [273, 274]. Conversely, TREM2 overexpression may mitigate mitochondrial damage and suppress inflammatory responses through the TREM2-Sirtuin3 (SIRT3)-NAD^+^ signaling axis [275, 276]. Additionally, missense variants in translocase of outer mitochondrial membrane 40 (TOMM40), a mitochondrial outer membrane protein, have been shown to induce mitochondrial dysfunction and trigger NLRP3-dependent inflammation [277], further underscoring the central role of mitochondrial integrity and neuroinflammation in the progression of AD.

Recent studies have further demonstrated that Aβ and Tau proteins can reciprocally exacerbate mitochondrial dysfunction and establish a positive feedback loop with the NLRP3 inflammasome [278–280]. Specifically, Aβ induces mitochondrial impairment and promotes the release of mtROS via the spleen tyrosine kinase (Syk)-AMPK signaling pathway, which subsequently activates the TRPM2 channel, leading to Ca^2 +^ influx and further amplification of the NLRP3-mediated inflammatory response and cognitive decline [16, 281]. The AGE-TXNIP axis facilitates Aβ accumulation in mitochondria and hyperactivates Drp1, competitively disrupting the hexokinase I (HK1)/VDAC complex, thereby triggering excessive mitochondrial fission and glycolytic dysregulation. This cascade ultimately results in NLRP3 inflammasome activation and IL-1β secretion, highlighting the interplay between neuroinflammation and metabolic disturbances [282–284]. Moreover, Aβ-induced mtDNA leakage can activate the mtDNA-STING-NLRP3/IL-1β axis and recruit neutrophils, thereby aggravating neuronal injury [285], indicating that Aβ may propagate neuroinflammatory processes through interactions with immune cells.

Given this mechanistic evidence, restoring mitochondrial homeostasis has emerged as a pivotal strategy for mitigating neuroinflammation in AD. Enhancing mitophagy represents a central component of this approach. It not only clears damaged mitochondria via the PINK1-Parkin pathway [286–289], thereby eliminating stimuli for NLRP3 activation, but also engages alternative regulatory pathways such as FOXO3a-Bcl2 interacting protein 3 (BNIP3) and SIRT1- high mobility group box 1 (HMGB1), which act synergistically to suppress NLRP3 inflammasome assembly [269, 290]. Thus, targeting mitochondrial quality control and functional integrity offers a promising therapeutic avenue for interrupting the self-perpetuating cycle of neuroinflammation in AD.

#### ERS driving NLRP3 inflammatory response in AD

Under the AD state, ERS activates the NLRP3 inflammasome through multiple signaling pathways, thereby exacerbating synaptic dysfunction and neurodegenerative changes [291, 292]. The specific mechanisms are as follows: First, the translocation of calreticulin (CALR) results in ER calcium overload, disrupts the homeostasis of the UPR, promotes Aβ deposition, and directly activates the NLRP3 inflammasome [292–294]. Second, in AD, ERS facilitates the assembly of the NLRP3 inflammasome and the release of IL-1β by activating the TXNIP pathway and the GSK3-NF-κB signaling cascade [291, 295]. It is noteworthy that the ERS pathway represents another critical mechanism by which the APOE allele modulates the NLRP3-mediated inflammatory response. In comparison to APOE3, APOE4 not only enhances MAMs function, but its proteolytic fragments also increase the expression of ER chaperone proteins such as GRP78, induce excessive phosphorylation of both tau and ERS-related proteins, and contribute to impaired axonal transport and neuroinflammation [296–298]. The ER-Golgi intermediate compartment (ERGIC) may serve as a quality control center for mutant TREM2 and has been identified in association with the mammalian target of rapamycin (mTOR) [299]. Dysregulation of the mTOR signaling pathway can trigger ERS [300], potentially exacerbating neuroinflammatory damage in AD through activation of the NLRP3 inflammasome. These mechanisms not only amplify neuroinflammation but also accelerate neuronal death and the pathological progression of AD via UPR dysregulation, calcium homeostasis disruption, and inflammatory cytokine storms. Therefore, targeting the ERS-NLRP3 axis may represent a critical strategy for intervening in neuroinflammation in AD.

#### Lysosomal stress driving NLRP3 inflammatory response in AD

In the pathogenesis of AD, dysfunction of the autophagy-lysosome pathway (ALP) plays a pivotal role in driving both the activation of the NLRP3 inflammasome and the deposition of pathological proteins via a dual mechanism. First, abnormal release of CTSB can directly trigger the activation of the NLRP3 inflammasome [301–303]. Second, the ALP modulates the AMPK/AKT signaling pathway to activate key transcription factors, including TFEB and transcription factor binding to IGHM enhancer 3 (TFE3), thereby promoting lysosome-mediated clearance of Aβ and Tau proteins while accelerating autophagic degradation of NLRP3 inflammasome components [304]. Conversely, glia maturation factor (GMF) exacerbates this process by amplifying NLRP3 inflammatory signaling and inhibiting autophagy, leading to a detrimental feedback loop characterized by “inflammation-autophagy imbalance” [305]. In contrast, certain branches of the ALP, such as the enhanced CMA pathway, have been demonstrated to effectively clear pathological proteins, suppress neuroinflammation, and preserve cognitive function [306]. Collectively, these mechanisms underscore the critical role of autophagy-lysosome dysfunction in linking inflammatory responses with protein homeostasis imbalances in AD pathology.

It is noteworthy that glial cells in AD can contribute to the clearance of pathological proteins such as Aβ and Tau through their lysosomal system [307, 308]. Among these, microglia utilize the autophagy-related protein P62, transcription factor TFEB, and the optineurin (OPTN)-microtubule-associated protein 1 light chain 3β II (MAP1LC3β II)-protein kinase RNA-activated kinase α1 (PRKA α1) pathway to enhance autophagic-lysosomal function, thereby improving the clearance of Aβ and NLRP3 inflammasomes [309–311]. However, when autophagy-lysosome dysfunction occurs in microglia and astrocytes, it not only exacerbates pathological protein deposition but may also accelerate AD progression by activating the NLRP3 inflammasome and inducing neuroinflammatory responses [307, 308]. The dual role of glial cells in pathological clearance and inflammatory activation highlights their complex regulatory network in AD and provides a critical entry point for developing novel therapeutic strategies targeting ALP-glial cell interactions.

Furthermore, the pathogenic mechanisms of AD mediated by the APOE and TREM2 genes are closely associated with dysfunction of the ALP. APOE4 can synergistically induce lysosomal δ-secretase leakage and activation in conjunction with 27-hydroxycholesterol (27-OHC), thereby promoting the production of Aβ and NFTs [312]. This process contributes to the accumulation of oxidized cholesterol, aberrant mammalian target of rapamycin complex 1 (mTORC1) activation, and disruption of lysosomal calcium homeostasis. Ultimately, these alterations lead to robust activation of NLRP3 inflammasomes through pathways involving CTSB release, thereby amplifying neuroinflammatory responses [313–315]. In contrast, the impaired binding of APOE2 to low-density lipoprotein receptors (LDLRs) may confer protective effects by reducing the sequestration of pathogenic lipids within lysosomes, potentially preventing lysosomal dysfunction-induced inflammation in AD [316]. Similarly, TREM2 plays a critical role in modulating microglial function. Upregulation of TREM2 via mTOR signaling has been shown to restore lysosomal function and enhance Aβ clearance [317], whereas the TREM2 mutation is linked to lysosomal impairment, which may exacerbate neuroinflammation [318]. Therefore, targeting the TREM2-organelle stress-NLRP3 inflammatory axis holds promise as a therapeutic strategy to promote the neuroprotective functions of microglia and mitigate neuroinflammation in AD.

### The organelle stress and NLRP3 inflammatory response regulation in PD

PD is a NDD primarily characterized by motor dysfunction. The core pathological features of PD include the degeneration of dopaminergic neurons in the substantia nigra pars compacta (SNpc) and a marked reduction in the level of dopamine (DA) in the striatum [319]. A hallmark pathological feature of PD is the abnormal accumulation of misfolded α-Syn within Lewy bodies and Lewy neurites [320]. Notably, α-Syn has been demonstrated to directly activate the NLRP3 inflammasome through inducing organelle dysfunction in an LPS-independent manner [321]. This suggests that the aberrant activation of the NLRP3 inflammasome during the progression of PD may result from the disruption of organelle homeostasis mediated by α-Syn.

#### Mitochondrial stress driving NLRP3 inflammatory response in PD

In PD, α-syn not only directly exacerbates mitochondrial damage in dopaminergic neurons, evidenced by reduced membrane potential, accumulation of mtDNA mutations, and abnormal oxygen consumption rates, but also induces oxidative phosphorylation dysfunction and disruption of redox homeostasis, ultimately driving neuronal degeneration via metabolic toxic pathways [322–324]. Concurrently, mitochondrial dysfunction in glial cells significantly contributes to the amplification of neuroinflammatory responses. In microglia, mitochondrial impairment promotes aberrant translocation of NLRP3 to mitochondria, leading to mitochondrial dynamic imbalance and oxidative stress, which directly activates NLRP3 inflammasomes and accelerates dopaminergic neuron degeneration [13, 325–329]. Moreover, deficient mitophagy in microglia and astrocytes results in the pathological accumulation of damaged mitochondria and ASC, thereby enhancing NLRP3-mediated inflammatory signaling and establishing a critical feedback loop that facilitates PD pathogenesis [330, 331].

Leucine-rich repeat kinase 2 (LRRK2) is a key protein kinase associated with PD, and its gain-of-function mutations, such as G2019S, R1441C, and G2385R, are among the most common genetic risk factors for PD [332]. These mutations disrupt mitochondrial and lysosomal homeostasis, thereby jointly promoting neurodegenerative processes [333–336]. Specifically, mutant LRRK2 activates the NF-κB inflammatory pathway and cleaves GSDMD, leading to the release of mtROS and mtDNA, which serve as potent activators of the NLRP3 inflammasome [337–339]. Additionally, mutant LRRK2 inhibits sarcoplasmic reticulum Ca^2 +^ -ATPase (SERCA), resulting in ER calcium depletion and subsequent mitochondrial calcium overload, ultimately impairing mitochondrial function [340, 341]. It also hyperphosphorylates Drp1, exacerbating mitochondrial fission while simultaneously impairing mitophagy, thus further amplifying mitochondrial dysfunction and sustaining NLRP3 inflammasome activation [342, 343].

#### ERS driving NLRP3 inflammatory response in PD

The degeneration of DA neurons is believed to be associated with neuroinflammation mediated by the NLRP3 inflammasome, which is driven by ERS [18]. Recent studies have demonstrated that inhibiting ERS can significantly enhance motor function and alleviate pathological damage in PD model animals, with the mechanism involving negative regulation of the NLRP3 signaling pathway [344]. Knockout of protein kinase C δ (PKCδ) in microglia suppresses α-Syn-induced ER-NLRP3 signaling and reduces IL-1β release [345]. Astrocytes can ameliorate the pathological progression of PD by upregulating uncoupling protein 2 (UCP2) or activating the ligand protein phoenixine-14 (PNX-14) of G protein-coupled receptor 173 (GPR173), thereby mitigating the NLRP3 inflammasome response associated with ERS [346, 347]. These findings indicate that targeting excessive activation of ERS to attenuate NLRP3 inflammatory signaling may represent a novel therapeutic direction for PD treatment.

#### Lysosomal stress driving NLRP3 inflammatory response in PD

Lysosomal dysfunction represents another pivotal mechanism contributing to disease progression in PD. Evidence indicates that LMP not only enhances the cellular uptake of α-syn fibrils but also mediates their intercellular propagation and soluble α-syn seeds formation via lysosome-dependent transfer, thereby accelerating disease spread throughout the brain [348]. Notably, α-syn can further exacerbate LMP, induce leakage of lysosomal contents, and impair ALP function, consequently activating the NLRP3 inflammasome and intensifying neurotoxicity in PD [349, 350]. In this process, mutant LRRK2 acts as a central regulator of the endolysosomal pathway. By hyperphosphorylating Rab GTPases such as RAB10, it compromises lysosomal activity, induces autophagic impairment and CTSB leakage, thereby directly activating the NLRP3 inflammasome and perpetuating a vicious cycle with α-syn aggregation [351, 352]. Additionally, α-syn accumulation disrupts vesicular trafficking, fusion, and recycling processes, ultimately compromising autophagolysosomal degradation pathways [17]. Intervention studies have confirmed that knocking out LRRK2 or overexpressing ATPase 13A2 (ATP13A2) in microglia can rescue lysosomal damage-induced NLRP3 inflammasome activation [351, 353]. Furthermore, activating cannabinoid receptor 2 (CB2R) in astrocytes enhances autophagic activity, promoting NLRP3 degradation and ameliorating pathological features in a mouse model of PD [354]. Collectively, these findings underscore the pivotal role of lysosomal homeostasis and autophagy in PD-associated neuroinflammation and highlight the therapeutic potential of targeting the lysosome-autophagy axis to modulate NLRP3-driven inflammatory signaling.

### The organelle stress and NLRP3 inflammatory response regulation in MS

MS is characterized by the progressive demyelination of the central nervous system, which serves as its primary pathological hallmark. When demyelination and neurodegenerative lesions impact both gray and white matter, severe neurological dysfunctions may arise, encompassing visual disturbances, muscle weakness, fatigue, cognitive deficits, and emotional impairments [355]. In the context of MS pathology, metabolic dysregulation in glial cells represents a central mechanism driving disease progression. This dysregulation establishes and reinforces a vicious cycle of neuroinflammation and demyelination via NLRP3 inflammasome activation. Notably, distinct glial cell types undergo characteristic metabolic reprogramming. Astrocytes exhibit heightened aerobic glycolysis, upregulated expression of the lactate transporter monocarboxylate transporter 4 (MCT4), and accelerated glycolytic flux and proliferation mediated by NF-κB and c-Myc signaling pathways. This metabolic shift not only compromises neuronal support functions but also disrupts metabolic homeostasis, thereby promoting NLRP3 activation, inflammatory infiltration, and demyelination [356, 357]. Upon polarization to the pro-inflammatory state, microglia adopt a metabolic profile dominated by glycolysis and glutaminolysis. Upregulation of glutaminase 1 (GLS1) enhances glutamine catabolism, further stimulating NLRP3 inflammasome activity and contributing to axonal degeneration [358, 359]. Oligodendrocytes, essential for myelin synthesis and maintenance [360], are particularly vulnerable to mitochondrial dysfunction, which leads to the accumulation of metabolic intermediates and ROS. These disturbances trigger NLRP3 inflammasome activation, exacerbating myelin breakdown and oligodendrocyte apoptosis, thereby directly amplifying demyelination in MS [361–364]. Collectively, metabolic impairments across glial cell populations not only compromise their intrinsic functions but also convert metabolic stress into sustained and destructive neuroinflammation through coordinated NLRP3 inflammasome activation, and ultimately promoting MS-associated neurodegeneration in a synergistic manner. Therefore, elucidating the critical regulatory mechanisms underlying myelin repair and neuroprotection in MS represents a key focus for the advancement of innovative therapeutic strategies.

#### Mitochondrial stress driving NLRP3 inflammatory response in MS

Mitochondrial dysfunction plays a critical role in demyelination and axonal degeneration in MS, particularly through aberrant mtDNA release and disrupted calcium homeostasis, both of which exacerbate oxidative and inflammatory damage in neurons and glial cells [365–367]. For instance, genetic deletion of vacuole membrane protein 1 (VMP1) has been shown to induce mitochondrial Ca^2 +^ overload and mtDNA release, thereby activating NLRP3 inflammasome [364]. Similarly, loss of SOD2 increases oxidative stress, leading to myelin breakdown and immune cell infiltration [368], whereas mitochondria-targeted antioxidants have demonstrated protective effects in animal models of MS [369]. On the other hand, the C-type lectin domain-containing gene 16A (CLEC16A), a susceptibility gene for MS, exerts protective effects by enhancing mitophagy and limiting the accumulation of damaged mitochondrial components, thereby suppressing NF-κB signaling, NLRP3 inflammasome activation, and GSDMD cleavage [357]. These findings collectively suggest that therapeutic strategies aimed at restoring mitochondrial ion homeostasis, enhancing autophagic clearance, and reducing the release of pathogenic mitochondrial contents may offer novel approaches for mitigating demyelination in MS.

#### Lysosomal stress driving NLRP3 inflammatory response in MS

Lysosomes play a dual regulatory role in MS. First, monomethyl fumarate (MMF) activates the lysosome-associated G protein-coupled receptor 109A (GPR109A), thereby enhancing lysosomal phagocytic function and reducing the levels of NLRP3 and IL-1β. This suggests that MMF may alleviate the progression of MS by inhibiting neuroinflammation [370]. Second, Epstein-Barr virus (EBV) employs its encoded G protein-coupled receptor BILF1 to induce ubiquitin-fold modifier 1 (UFM1)-dependent ubiquitination of MAVS. This modification induces the translocation of MAVS from the outer mitochondrial membrane to mitochondria-derived vesicles (MDVs), where it is subsequently encapsulated and ultimately degraded by lysosomes. This process enables EBV to evade host immune surveillance by blocking innate immune signaling mediated by MAVS and inhibiting the activation of the NLRP3 inflammasome, thereby exacerbating the pathological damage associated with MS [371]. These findings indicate that precise regulation of lysosomal degradation may represent a novel therapeutic strategy for MS intervention.

### The organelle stress and NLRP3 inflammatory response regulation in ALS

ALS is a progressive NDD affecting both upper and lower motor neurons, characterized by increased cortical excitability. This condition can lead to neuronal dysfunction in the bulbar, cervical, thoracic, or lumbar regions of the spinal cord [372]. Despite its relatively low incidence, ALS progresses rapidly to its terminal stage, resulting in severe motor dysfunction and ultimately death. Research has demonstrated that the pathogenesis of ALS is closely associated with mitochondrial dysfunction. For instance, poly-glycine-alanine droplet protein (GA-DRP) synergizes with sulfide quinone oxidoreductase (SQOR) to promote the aberrant synthesis and release of mtDNA and mtROS. This process activates the NLRP3 inflammasome, exacerbates neuroinflammation, and accelerates the pathological progression of ALS [373]. These findings indicate that targeted modulation of mitochondrial homeostasis may represent a pivotal therapeutic strategy for ALS intervention.

### The organelle stress and NLRP3 inflammatory response regulation in HD

HD is a NDD characterized by dystonia, dyskinesia, cognitive impairment, and abnormal behavior. The core pathological mechanism involves neuronal damage mediated by the mutant Huntingtin protein (mHTT). Research has demonstrated that Gal3 exacerbates the progression of HD by inhibiting ubiquitination modifications of key proteins in damaged lysosomes, suppressing the autophagy clearance pathway, and inducing the NLRP3 inflammasome response, thereby leading to the abnormal aggregation of mHTT [374]. These findings suggest that targeted modulation of Gal3 or restoration of autophagy-lysosome function may represent potential therapeutic strategies for the management of HD.

**Fig. 5:**
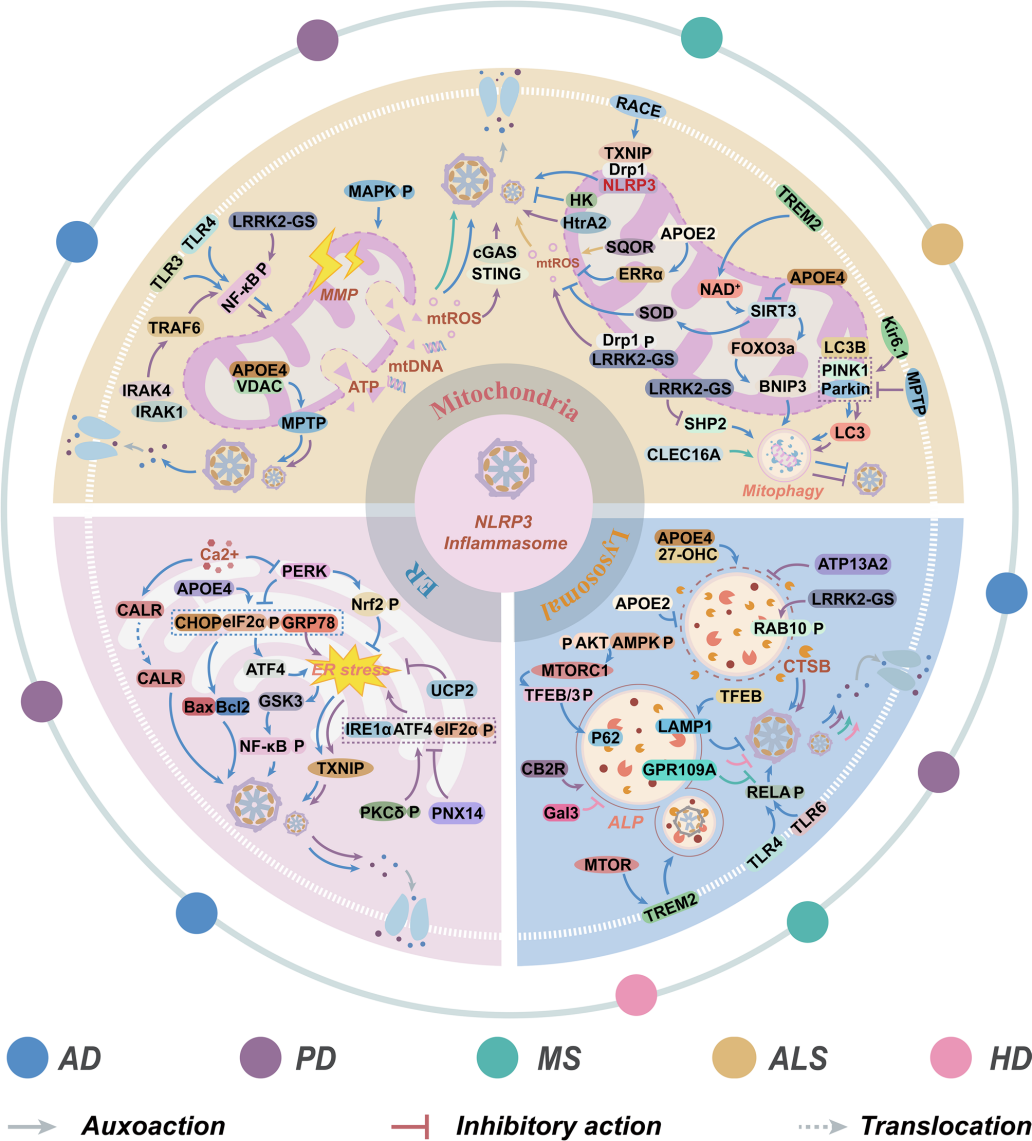
The role of organelles stress in driving NLRP3 inflammatory response in NDDs. Mitochondrial stress activates the NLRP3 inflammasome via signaling pathways such as NF-κB, MAPK, cGAS-STING, and TXNIP, thereby exacerbating AD, PD, MS, and ALS. Additionally, mechanisms such as SIRT3–FOXO3a-BNIP3, LC3B-PINK1-Parkin, and SHP2 promote mitophagy to degrade the NLRP3 inflammasome, thereby alleviating the pathological progression of AD and PD. The ER releases Ca^2+^, inducing CALR translocation and ERS, and activates the NLRP3 inflammasome through pathways such as Bax-Bcl2, NF-κB, and TXNIP, further aggravating AD and PD. Lysosomal damage leads to the release of CTSB, which drives the NLRP3 inflammatory response in AD and PD. Conversely, ALP inhibits the NLRP3 inflammatory response in AD, MS, and HD. Abbreviations: APOE, apolipoprotein E; ATF4, activating transcription factor; AKT, protein kinase B; ALP, autophagy-lysosome pathway; AMPK, AMP-activated protein kinase; ATP13A2, ATPase 13A2; Bax, Bcl2-associated X protein; Bcl2, B-cell lymphoma 2; BNIP3, Bcl2 interacting protein 3; CALR, Calreticulin; CB2R, cannabinoid receptor 2; cGAS, cyclic GMP-AMP synthase; CHOP, C/EBP homologous protein; CLEC16A, C-type lectin domain-containing gene 16A; CTSB, cathepsin B; Drp1, dynamin-related protein 1; eIF2α, eukaryotic initiation factor 2α; ERRα, estrogen-related receptor α; FOXO3a, Forkhead box protein O3a; Gal3, Galectin 3; GPR109A, G protein-coupled receptor 109A; GRP78, glucose-regulated protein 78; GSK3, glycogen synthase kinase 3; HK, Hexokinase; IRAK, interleukin-1 receptor-associated kinase; IRE1α, inositol-requiring enzyme 1α; LAMP1, lysosomal-associated membrane protein 1; LC3, microtubule-associated protein light chain 3; LRRK2-GS, leucine-rich repeat kinase 2-G2019S; MMP, mitochondrial membrane potential; MPTP, mitochondrial permeability transition pore; mtDNA, mitochondrial DNA; mtROS, mitochondrial reactive oxygen species; MTOR, mammalian target of rapamycin; MTORC1, mammalian target of rapamycin complex 1; NF-κB, nuclear factor kappa B; NLRP3, the NOD-like receptor family pyrin domain-containing 3; Nrf2, nuclear factor erythroid 2-related factor 2; 27-OHC, 27-hydroxycholesterol; P62, sequestosome-1; PERK, protein kinase R-like er kinase; PINK1, PTEN-induced kinase 1; PNX14, phoenixin 14; PKCδ, protein kinase C δ; RAB10, ras-related protein 10; RACE, rapid-amplification of cDNA ends; RELA, V-rel reticuloendotheliosis viral oncogene homolog a; SHP2, src homology 2 domain-containing protein tyrosine phosphatase 2; SIRT3, silent information regulator 3; SOD, superoxide dismutase; STING, stimulator of interferon genes; SQOR, sulfide quinone oxidoreductase; TFEB, transcription factor EB; TLR, toll-like receptor; TRAF6, recombinant TNF receptor-associated factor 6; TREM2, triggering receptor expressed on myeloid cells 2; TXNIP, thioredoxin-interacting protein; UCP2, uncoupling protein 2; VDAC, voltage-dependent anion channel

In the pathogenesis of NDDs, organelle stress and its dynamic interaction network serve as the central driving force for disease progression by modulating the activation of the NLRP3 inflammasome (Fig. 5). For instance, in AD, mitochondrial dysfunction, characterized by the accumulation of mtROS, establishes a bidirectional interaction with Aβ or Tau pathology. This interaction not only activates the NLRP3 inflammasome but also exacerbates neuroinflammation. ERS triggers NLRP3 activation via calcium overload or TXNIP signaling, whereas lysosomal autophagy dysfunction induces inflammation through CTSB leakage. In PD, α-Syn induces inflammation by promoting mitochondrial-localized NLRP3 activation, ERS-PKCδ signaling, and LMP. MS and ALS are associated with mitochondrial Ca^2 +^ homeostasis imbalance, mtDNA leakage, or SQOR-mediated mtROS release, all of which contribute to the activation of NLRP3 inflammatory pathways. In HD, Gal3-mediated inhibition of autophagy-lysosome facilitates mHTT aggregation and inflammatory responses. Consequently, therapeutic intervention aimed at the crosstalk between organelle homeostasis and the NLRP3-driven inflammatory response may offer novel strategies for NDDs by disrupting the vicious cycle from organellar dysfunction to progressive pathology.

## Potential therapeutic agents targeting organelles stress and NLRP3 inflammatory response

In NDDs, aberrant activation of the NLRP3 inflammasome and dysfunction of multiple organelles constitute a self-reinforcing pathological loop. This synergistic dysregulation mechanism offers a promising therapeutic target for targeted interventions. By leveraging advanced technologies such as natural compounds and nanodelivery systems, precise regulation of the organelle–NLRP3 communication axis is anticipated to inhibit neuroinflammation at its source, thereby opening new avenues for therapeutic intervention.

### Potential therapeutic agents targeting mitochondria-NLRP3 axis

Natural medicines and their active constituents have demonstrated considerable potential in mitigating neuroinflammation associated with NDDs through mitochondrial targeting. In AD research, the traditional Chinese medicine formulation Kai Xin San, along with various plant-derived compounds, such as 2,3,5,4’-tetrahydroxystilbene-2-O-β-D-glucoside (TSG) from Polygonum multiflorum, cornuside, and ciliatoside A (CA) from Peristrophe japonica, have been shown to suppress the NLRP3 inflammasome activation by promoting PINK1-Parkin pathway-dependent mitophagy [286–289]. While andrographolide triacetate (ADA) relies on the SIRT3–FOXO3a signaling pathway to induce non-PINK1-Parkin-dependent mitochondrial autophagy, thereby clearing the NLRP3 inflammasome and revealing a multi-pathway synergistic mechanism for mitochondrial quality control [269]. Beyond enhancing mitophagy, the natural flavonoid kaempferol (Myr) attenuates NLRP3 activation linked to mitochondrial dysfunction by inhibiting the p38 MAPK signaling pathway, thereby restoring mitochondrial dynamics and biogenesis and delaying AD progression [15]. In PD models, andrographolide (Andro) activates the PINK1-Parkin pathway to facilitate mitophagy [329], while Dl-3-n-butylphthalide (NBP), derived from celery seeds, suppresses the PARP1-poly(ADP-ribose) (PAR) pathway, effectively inhibiting NLRP3 inflammasome activation [325]. In ALS-related neuroinflammation, irisflorentin (IFR) inhibits glycine-alanine dipeptide repeat proteins (GA-DPRs)-sulfide quinone oxidoreductase (SQOR) signaling pathway, reducing mtDNA leakage and mtROS production, thus preventing NLRP3 inflammasome activation [373].

In addition to natural medicines, research into new indications for certain marketed drugs has provided novel insights into the treatment of NDDs. For example, the non-steroidal anti-inflammatory drug flufenamic acid has been shown to inhibit NLRP3 inflammasome activation induced by mitochondrial dysfunction through modulation of the Syk-AMPK axis [16]. Meanwhile, the calcium channel blocker verapamil exerts similar effects via the RAGE-TXNIP pathway, offering multi-target intervention strategies for AD [282]. Fingolimod (FTY720) attenuates mtROS production by suppressing the PI3K/AKT/GSK-3β signaling pathway and thereby limits NLRP3-mediated neuroinflammation in PD [328]. Furthermore, a range of mitochondria-targeted antioxidants have demonstrated therapeutic potential by specifically addressing mitochondrial oxidative stress. Compounds such as TSC, assembled from superoxide dismutase, catalase, and tannic acid, as well as mitoquinone (MitoQ), effectively scavenge excessive mtROS, thereby inhibiting NLRP3 inflammasome activation [375, 376]. Recent studies also indicate that mitochondria-targeted antioxidants may hold promise for the treatment of MS [369]. Although their precise efficacy requires further validation, when combined with recently developed organelle-specific nanodelivery systems, these agents may significantly improve drug targeting and bioavailability, potentially opening new avenues for the treatment of NDDs.

### Potential therapeutic agents targeting ER-NLRP3 axis

In NDDs, ERS serves as a critical mechanism underlying the aberrant activation of the NLRP3 inflammasome. Excessive accumulation of cytoplasmic Ca^2+^ shifts the UPR toward pro-apoptotic signaling, thereby promoting NLRP3 inflammasome activation and facilitating Aβ seed formation. Meanwhile, liposomal nanomedicine Felodipine@LND, encapsulating the calcium channel blocker felodipine, is capable of crossing the blood-brain barrier, inhibiting the PERK-eIF2α-ATF4 signaling axis, and exerting anti-neuroinflammatory effects in AD [293]. Additionally, salubrinal (SAL), a selective inhibitor of eIF2α, attenuates ERS-mediated NLRP3 activation via suppression of the α-Syn-TXNIP-Trx axis, demonstrating therapeutic potential in PD models [345]. Moreover, the PI3K-AKT-GSK3β pathway is critically involved in ERS-driven NLRP3 inflammasome activation, and punicalagin (PUN) has been shown to ameliorate PD pathology by targeting this cascade [344].

### Potential therapeutic agents targeting lysosome-NLRP3 axis

ALP is a critical cellular mechanism responsible for the clearance of abnormal protein aggregates and the regulation of inflammatory responses. Its dysfunction has been closely associated with aberrant inflammatory activation in NDDs. In PD research, Ganoderma lucidum extract (GLE) has been demonstrated to target the iNOS-NLRP3 signaling pathway, effectively suppressing neuroinflammation induced by impaired lysosomal degradation [350]. Future studies may focus on the precise modulation of the core ALP pathway and, in conjunction with emerging nano-targeted delivery systems, develop multifunctional therapeutic agents capable of synergistically enhancing autophagic flux and inhibiting pro-inflammatory pathways. Such strategies could pave the way for more efficient and targeted therapeutic interventions in NDDs.

In summary, strategies that target organelle homeostasis to control NLRP3 inflammasome activation represent a promising direction for treating NDDs. A range of agents, from natural products to repurposed drugs and mitochondrial antioxidants, show efficacy in breaking the cycle of neuroinflammation by improving organellar function (Table 2). Although some of these approaches are still exploratory, their integration with emerging nanodelivery platforms holds great potential to precisely regulate the organelle-NLRP3 axis, thereby opening novel and promising therapeutic avenues for NDDs.

**Table 2:** Potential therapeutic agents targeting the organelle-NLRP3 inflammatory axis for the intervention of NDDs

Drug	Stage	Target/Pathway	Outcome on NLRP3	NDD	Reference
Kai Xin San	clinical	LC3B-PINK1-Parkin	Activators	AD	[288]
TSG	clinical	AMPK-PINK1-Parkin		AD	[286]
Cornuside	preclinical	PINK1-Parkin		AD	[287]
CA	preclinical	AMPK-PINK1-Parkin		AD	[289]
ADA	preclinical	SIRT3–FOXO3a		AD	[269]
Andro	preclinical	PINK1-Parkin		PD	[329]
Myr	preclinical	P38 MAPK	Inhibitors	AD	[15]
Flufenamic acid	preclinical	Syk-NLRP3		AD	[16]
Verapamil	preclinical	RAGE-TXNIP		AD	[282]
NBP	clinical	PARP1-PAR		PD	[325, 377]
FTY720	preclinical	PI3K-AKT-GSK 3β		PD	[328]
IFR	preclinical	GA-DPR-SQOR		ALS	[373]
Felodipine@LND	preclinical	PERK-EIFα-ATF4	Inhibitors	AD	[293]
SAL	preclinical	αSyn-TXNIP-Trx		PD	[345]
PUN	preclinical	PI3K-AKT-GSK 3β		PD	[344]
GLE	clinical	iNOS-NLRP3	Inhibitors	PD	[350]

Drug names are not marked with different colors, as follows: Red: Kai Xin San, TSG, Cornuside, CA, ADA, Andro, Myr, Flufenamic acid, Verapamil, NBP, FTY720, IFR. Blue: Felodipine@LND, SAL, PUN. Brown: GLE

## Conclusion and prospect

The NLRP3 inflammasome, as a central regulatory hub of innate immunity, plays a pivotal role in the neuroinflammatory processes of NDDs by integrating multi-organelle stress signals and dynamic interaction networks. This review systematically elaborates on the intricate regulatory network of the NLRP3 inflammasome, which is orchestrated by multiple organelles, such as mitochondria and ER, through diverse mechanisms. These mechanisms encompass the release of stress signaling molecules, regulation of NLRP3 subcellular localization and PTMs, and provision of platforms for inflammasome assembly. Importantly, these organelles do not operate in isolation; rather, they form interaction networks via dynamic membrane contact sites and material exchange, enabling the integration and amplification of inflammatory signals that ultimately drive NLRP3 activation. In NDDs, the aggregation of pathological proteins becomes intricately intertwined with organelle dysfunction. This interaction fuels a self-perpetuating cycle of NLRP3 inflammasome activation and progressive pathological deterioration, thereby accelerating disease progression. We further summarize a range of potential therapeutic strategies targeting the organelle-NLRP3 axis, including natural compounds, drug repurposing approaches, and mitochondria-targeted antioxidants. These interventions demonstrate considerable neuroprotective potential in preclinical models by restoring organelle homeostasis, enhancing quality control processes, or directly inhibiting the NLRP3 pathway. Currently, intervention strategies for diseases such as AD and PD have evolved from merely inhibiting NLRP3 to multidimensional regulation of organelle homeostasis. For example, TSG promotes the elimination of damaged mitochondria by activating PINK1-Parkin-mediated mitochondrial autophagy, highlighting the potential of multi-target synergistic therapy.

However, numerous critical scientific challenges in this field remain to be urgently addressed. First, the dynamic regulatory mechanism underlying the subcellular localization of NLRP3 has yet to be fully elucidated, particularly its transport rules and pathological significance among organelles such as mitochondria and the Golgi apparatus under disease conditions. Second, the cooperative regulatory networks among different organelles exhibit significant disease specificity. For instance, in AD, Aβ deposition primarily disrupts mitochondrial-lysosomal interactions, whereas in PD, α-Syn tends to impair ER-Golgi apparatus transport systems. The molecular basis for this heterogeneity still requires systematic investigation. Moreover, current therapeutic strategies remain largely limited to modulating individual organelles, while precise targeting of inter-organelle communication still poses significant technical challenges. A deeper understanding of the cell-type-specific heterogeneity of the organelle-NLRP3 axis is required to lay the groundwork for novel precision interventions. Future studies should aim to construct an NDDS-specific organelle interaction map to uncover the causal relationship between pathological protein aggregation and the organelle stress network. Therapeutic strategies should emphasize a dual regulatory approach that not only sustains the homeostasis of key organelles but also inhibits inflammatory cascades triggered by their abnormal interactions. By thoroughly analyzing the dynamic interaction mechanisms between the organelle network and the NLRP3 inflammasome, new avenues for the precise intervention of NDDs will be opened.

## Data Availability

No datasets were generated or analysed during the current study.

## Abbreviations

Aβ: β-Amyloid
ABC: ATP-binding cassette transporter
ABHD5: Abhydrolase domain containing 5
AD: Alzheimer’s Disease
ADA: Andrographolide triacetate
ADCY7: Adenylate cyclase 7
AIS: Acute ischemic stroke
AKI: Acute kidney injury
AKT: Protein kinase B
ALDOA: Fructose-bisphosphate aldolase A
ALI: Acute lung injury
ALOX12: Arachidonate-12-lipoxygenase
ALP: Autophagy-lysosome pathway
ALS: Amyotrophic lateral sclerosis
AMPK: AMP-activated protein kinase
Andro: Andrographolide
ANXA2: Annexin A2
APOE: Apolipoprotein E
ARDS: Acute respiratory distress syndrome
ASC: Apoptosis-associated speck-like protein containing CARD
ATF: Activating transcription factor
ATP13A2: ATPase 13A2
Bax: Bcl2-associated X protein
BE: Bacterial endotoxin
BNIP3: Bcl2 interacting protein 3
BRCC3: BRCA1-associated ring domain protein 3
CA: Ciliatoside A
CALR: Calreticulin
CaMK II: Calcium/calmodulin-dependent protein kinase II
Caspase-1: Cysteine aspartate-specific protease-1
CB2R: Cannabinoid receptor 2
CCs: Cholesterol crystals
Cd: Cadmium
c-di-AMP: Cyclic deadenylate
cGAS: Cyclic GMP-AMP synthase
CHOP: C/EBP homologous protein
CGRP: Calcitonin gene-related peptide
CLEC16A: C-type lectin domain-containing gene 16A
CLIC: Chloride intracellular channel
CMA: Chaperone-mediated autophagy
CMPK2: Cytidine/uridine monophosphate kinase 2
COPD: Chronic obstructive pulmonary disease
CS: Crystalline Silica
CTS: Cathepsin
CVST: Cerebral venous sinus thrombosis
CXCL4: C-X-C motif chemokine 4
CYLD: Cylindromatosis
Cytc: Cytochrome c
DA: Dopamine
DAMPs: Damage-associated molecular patterns
2-DG: 2-Deoxy-D-Glucose
DRP1: Dynamin-related protein 1
DRPs: Dipeptide repeat proteins
dsDNA: Cytosolic double-stranded DNA
dTGN: Dispersed trans-Golgi network
eATP: Extracellular ATP
EBV: Epstein-Barr virus
EECS: Endoplasmic reticulum-endosome membrane contact sites
eIF2α: Eukaryotic initiation factor 2α
EMP: Embden-Meyerhof-Parnas
ERGIC: ER-Golgi intermediate compartment
ERRα: Estrogen-related receptor α
ERS: Endoplasmic reticulum stress
ETT: Endosome-to-trans-Golgi network trafficking
FA: Folic acid
FABP: Fatty acid binding protein
FAD: Flavin adenine dinucleotide
FAM3A: Family with sequence similarity 3-member A
FASN: Fatty acid synthase
FliI: Flightless I
FOXO3a: Forkhead box protein O3a
FTD: Frontotemporal dementia
FTY720: Fingolimod
GABA: Gamma-aminobutyric acid
GA-DRP: Glycine-alanine dipeptide repeat protein
Gal: Galectin
GBF1: Golgi-specific brefeldin A-resistance factor 1
GBP: Guanylate-binding protein
GLE: Ganoderma lucidum extract
GLS1: Glutaminase 1
GLUT1: Glucose transporter 1
GMF: Glia maturation factor
GOLPH3: Golgi phosphoprotein 3
GPR: G protein-coupled receptor
GRP: Glucose-regulated protein
GSDMD: Gasdermin D
GSDMD-NT: GSDMD N-terminal domain
GSK: Glycogen synthase kinase
Hcy: Homocysteine
HD: Huntington’s disease
HDPE: High-density polyethylene
HFD: High-fat diet
HIF-1α: Hypoxia-inducible factor-1α
HK: Hexokinase
HO-1: Heme oxygenase-1
HS: Hidradenitis suppurativa
HTV: High tidal volume
IFNβ: Interferon-β
IFNAR: Interferon-α/β receptor 1
IFN-I: Type I interferon
IFR: Irisflorentin
IKKε: Inhibitor of NF-kB kinase ε
IL: Interleukin
iNOS: Inducible nitric oxide synthase
IP3R: Inositol 1,4,5-trisphosphate receptor
IRAK: Interleukin-1 receptor-associated kinase
IRE1α: Inositol-requiring enzyme 1α
IRF: Interferon regulatory factor
IRGM: Immunity-related GTPase M
IVDD: Intervertebral disc degeneration
JNK: c-Jun N-terminal kinase
KAT5: Lysine acetyltransferase 5
Keap1: Kelch-like ECH-associated protein 1
LAMP: Lysosomal-associated membrane protein
LC3: Microtubule-associated protein light chain 3
LCD: Lysosome-dependent cell death
LDLRs: Low-density lipoprotein receptors
LMN: Lower motor neuron
LMP: Lysosomal membrane permeabilization
LPC: Lysophosphatidylcholine
LPS: Lipopolysaccharide
LRRK2: Leucine-rich repeat kinase 2
LRRK2-GS: Leucine-rich repeat kinase 2-G2019S
LSD1: Lysine specific demethylase 1
LXRβ: Liver X receptor β
MAC: Complement membrane attack complex
MAMs: Mitochondria-associated membranes
MAPK: Mitogen-activated protein kinase
MAP1LC3β II: Microtubule-associated protein 1 light chain 3β II
MAPT: Hyperphosphorylated microtubule-associated protein tau
MARK4: Microtubule affinity-regulating kinase 4
MAVS: Mitochondrial antiviral signaling protein
MCSs: Membrane contact Sites
MCT4: Monocarboxylate transporter 4
MCU: Mitochondrial calcium uniporter
MDVs: Mitochondria-derived vesicles
MERCs: Mitochondria-endoplasmic reticulum contact sites
MFN1: Mitofusin 1
mHTT: Mutant Huntingtin protein
MIA: Monosodium iodoacetate
MitoQ: Mitoquinone
MMF: Monomethyl fumarate
MMP: Mitochondrial membrane potential
Mo: Molybdenum
MOMP: Mitochondrial outer membrane permeabilization
MPTP: Mitochondrial permeability transition pore
MS: Multiple sclerosis
MSU: Monosodium urate
mtDNA: Mitochondrial DNA
MTOC: Microtubule-organizing center
mTOR: Mammalian target of rapamycin
mTORC1: Mammalian target of rapamycin complex 1
mtROS: Mitochondrial reactive oxygen species
MYD88: Myeloid differentiation primary response protein 88
MYO18A: Myosin XVIIIA
Myr: Myricetin
NAFLD: Nonalcoholic fatty liver disease
NAMPs: Nanoparticle-associated molecular patterns
NBP: Dl-3-n-butylphthalide
NDDs: Neurodegenerative diseases
NEK7: NIMA-related kinase 7
NFATc1: Nuclear factor of activated T cells cytoplasmic 1
NF-κB: Nuclear factor kappa B
NFT: Neurofibrillary tangles
NIK: NF-κB-inducing kinase
NKAα1: Na^+^/K^+^ -ATPase α1
NLRP3: The NOD-like receptor family pyrin domain-containing 3
NOX2: NADPH oxidase 2
NPC1: Niemann-pick type C1 protein
NRF2: Nuclear factor erythroid 2-related factor 2
NS: Nephrotic syndrome
OA: Osteoarthritis
27-OHC: 27-hydroxycholesterol
OMV: Outer membrane vesicle
OPA1: Mitochondrial dynamin-like GTPase
OPTN: Optineurin
OSA: Obstructive sleep apnea
oxLDL: Oxidized low-density lipoprotein
Ox-mtDNA: Oxidized-mitochondrial DNA
OXPHOS: Oxidative phosphorylation
PA: Palmitate
PAMPs: Pathogen-associated molecular patterns
PAR: Poly (ADP-ribose)
PARP1: Poly (ADP-ribose) polymerase 1
PCM: Pericentriolar material
PCNT: Pericentrin
PD: Parkinson’s disease
PERK: Protein kinase R-like ER kinase
PGC-1α: Peroxisome proliferator-activated receptor gamma coactivator 1α
PI3K: Phosphatidylinositol 3-kinase
PINK1: PTEN-induced kinase 1
PI4P: Phosphatidylinositol 4-phosphate
PK: Protein kinase
PKM2: Pyruvate kinase M2
PLA2G7: Phospholipase A2 group VII
PLK: Polo-like kinase
PMP: Phagolysosomal membrane permeability
PNPT1: Polynucleotide phosphorylase 1
PNX-14: Phoenixin-14
POCD: Postoperative cognitive dysfunction
Poly GA: Poly glycine-alanine
PRKA α1: Protein kinase RNA-activated kinase α1
Pro: Precursor
PRR: Pattern recognition receptor
PTM: Post-translational modification
PTPIP51: Protein tyrosine phosphatase-interacting protein 51
PUN: Punicalagin
P2X7R: P2X purinergic receptor 7
Rab: Ras-related protein
RACE: Rapid-amplification of cDNA ends
RELA: V-rel reticuloendotheliosis viral oncogene homolog A
RNF34: Ring finger protein 34
Rubicon: Run domain protein as Beclin-1-interacting and cysteine-rich containing
SAL: Salubrinal
SCAP: Sterol regulatory element-binding protein cleavage-activating protein
SERCA: Sarcoplasmic reticulum Ca^2^^+^ -ATPase
SH: Small hydrophobic protein
SHP2: Src homology 2 domain-containing protein tyrosine phosphatase 2
SIC: Sepsis-induced cardiomyopathy
SIRT: Silent information regulator
SNpc: Substantia Nigra pars compacta
SNX10: Sorting nexin 10
SOD2: Superoxide dismutase 2
SPATA2: Surfactant-associated protein A2
SPs: Silicon dioxide particles
SQOR: Sulfide quinone oxidoreductase
SREBP2: Sterol regulatory element-binding protein 2
SS: Sodium sulfite
STAT3: Signal transducer and activator of transcription 3
STING: Stimulator of interferon genes
Stx2: Shiga toxin 2
STX17: Syntaxin 17
SOD2: Superoxide dismutase 2
STZ: Streptozotocin
SubAB: Subtilase cytotoxin
TAK1: Transforming growth factor-β-activated Kinase 1
TBK1: TANK binding kinase 1
TCA: Tricarboxylic acid
TF: Transcription factor
TFE3: Transcription factor binding to IGHM enhancer 3
TFEB: Transcription factor EB
TGN: Trans-Golgi network
TLR: Toll-like receptor
TME: Tumor microenvironment
TMEM176B: Transmembrane protein 176B
TOMM40: Translocase of outer mitochondrial membrane 40
TRAF6: Recombinant TNF receptor-associated factor 6
TREM2: Triggering receptor expressed on myeloid cells 2
TRIF: TIR domain-containing adapter-inducing interferon-β
TRIM59: Tripartite motif-containing 59
Trx: Thioredoxin
TSG: 2,3,5,4’-Tetrahydroxyphenyl-2-O-β-D-Glucoside
γ-TuRC: γ-tubulin ring complex
TXNIP: Thioredoxin-interacting protein
UCP2: Uncoupling protein 2
UFM1: Ubiquitin-fold modifier 1
ULK1: Unc-51-like autophagy activating kinase 1
UMN: Upper motor neuron
UPR: Unfolded protein response
VAMP8: Vesicle-associated membrane protein 8
VAPB: Vesicle-associated membrane protein-associated protein B
VDAC: Voltage-dependent anion channel
VEGF: Vascular endothelial growth factor
VILI: Ventilator-induced lung injury
VMP1: Vacuole membrane protein 1
XOD: Xanthine oxidase
ZDHHC: Zinc finger DHHC-type palmitoyl transferase family
ZFYVE21: Zinc finger FYVE-type containing 21
ZRR: ZFYVE21-Rubicon-RNF34
